# Functional relationship between mTERF4 and GUN1 in retrograde signaling

**DOI:** 10.1093/jxb/erv525

**Published:** 2015-12-18

**Authors:** Xuwu Sun, Duorong Xu, Zhixin Liu, Tatjana Kleine, Dario Leister

**Affiliations:** Plant Molecular Biology (Botany), Department Biology I, Ludwig-Maximilians-University Munich, 82152 Martinsried, Germany

**Keywords:** Chloroplast, GUN1, mTERF4/COE1, PGE, retrograde signaling.

## Abstract

Plastid-to-nucleus retrograde signaling plays an important role in regulating the expression of photosynthesis-associated nuclear genes (PhANGs) in accordance with physiological demands on chloroplast biogenesis and function. Despite its fundamental importance, little is known about the molecular nature of the plastid gene expression (PGE)-dependent type of retrograde signaling. PGE is a multifaceted process, and several factors, including pentatricopeptide repeat (PPR) proteins, are involved in its regulation. The PPR protein GUN1 plays a central role in PGE-dependent retrograde signaling. In this study, we isolated a mutant exhibiting up-regulation of *CHLOROPHYLL A/B-BINDING PROTEIN* (*CAB*) under normal growth conditions (named *coe1* for CAB overexpression 1). The *coe1* mutant has a single-base mutation in the gene for mitochondrial transcription termination factor 4 (mTERF4)/BSM/RUG2, which plays a role in regulating the processing of certain plastid transcripts. Defects in GUN1 or mTERF4 de-repressed the expression of specific plastid mRNAs in the presence of lincomycin (LIN). In wild-type plants, treatment with LIN or spectinomycin (SPE) inhibited processing of plastid transcripts. Comparative analysis revealed that in *gun1* and *coe1/mterf4*, but not in wild-type, *gun4*, or *gun5* plants, the processing of plastid transcripts and expression levels of *Lhcb1* mRNA were affected in opposite ways when plants were grown in the presence of LIN or SPE. In addition, the *coe1* mutation affected the intracellular accumulation and distribution of GUN1, as well as its plastid signaling activity. Taken together, these results suggest that GUN1 and COE1 cooperate in PGE and retrograde signaling.

## Highlight

Identification and characterization of mitochondrial transcription termination factor 4 (mTERF4), which cooperates with GUN1 to regulate plastid gene expression and plastid retrograde signaling.

## Introduction

Chloroplasts evolved from a free-living cyanobacterium, following its endosymbiotic integration into a non-photosynthetic eukaryotic host (Douglas and Raven, 2003). However, a large amount of genetic information has been transferred to the nuclear genome during the evolution of chloroplasts (Abdallah *et al.*, 2000; Rujan and Martin, 2001). At present, the plastid genome of higher plants contains only about 100 genes, which encode proteins for plastid gene expression (PGE) and photosynthesis (Abdallah *et al.*, 2000). In contrast, more than 95% of chloroplastic proteins are now encoded in the nucleus, translated in the cytoplasm, and then imported into the organelle (Barbrook *et al.*, 2006). Having been imported into the chloroplast, some nucleus-encoded plastid proteins interact with chloroplast-encoded proteins to form multi-subunit complexes involved in, for instance, the replication and expression of plastid-encoded genes, or in photosynthesis. To ensure correct stoichiometric assembly of these multi-subunit complexes, and enable their reorganization in response to developmental or environmental cues, the activities of the nuclear and chloroplast genomes must be coordinated through an intracellular signaling network (Kleine *et al.*, 2009; Grimm *et al.*, 2014). This network includes signaling pathways that originate in the nucleus (anterograde signaling) and the plastids (retrograde signaling) (Grimm *et al.*, 2014). Anterograde signaling is involved mainly in the regulation of PGE (Jiao *et al.*, 2007). By contrast, retrograde signaling conveys information about the developmental and metabolic state of the chloroplast to the nucleus, modifying nuclear gene expression in accordance with the current status of the organelle (Nott *et al.*, 2006; Kleine *et al.*, 2009; Pfannschmidt, 2010; Chi, *et al.*, 2013). Plastid signals are classified into five distinct groups, depending on their source: (i) PGE; (ii) tetrapyrrole biosynthesis; (iii) reactive oxygen species generation; (iv) plastid redox state; and (v) metabolites, such as 3-phosphoadenosine 5′-phosphate, methylerythritol cyclodiphosphate, and β-cyclocitral (Gray *et al.*, 2003; Baier and Dietz, 2005; Pesaresi *et al.*, 2007; Mochizuki *et al.*, 2008; Moulin *et al.*, 2008; von Gromoff *et al.*, 2008; Pfannschmidt *et al.*, 2009; Bräutigam *et al.*, 2009; Voigt *et al.*, 2010; Galvez-Valdivieso and Mullineaux, 2010; Estavillo *et al.*, 2011; Woodson *et al.*, 2011; Ramel *et al.*, 2012; Xiao *et al.*, 2012; Kim and Apel, 2013; Terry and Smith, 2013).

Tetrapyrrole biosynthesis and PGE-dependent signaling are the best characterized types of plastid signaling (Nott *et al.*, 2006). Much information on their operation has been obtained from studies on *gun* (genomes uncoupled) mutants of *Arabidopsis thaliana* (Nott *et al.*, 2006). Six independent *gun* mutants have been identified. Five of them (*gun2*–*gun6*) are defective in different steps in the tetrapyrrole biosynthetic pathway (Susek *et al.*, 1993; Mochizuki *et al.*, 2001; Larkin *et al.*, 2003; Strand *et al.*, 2003; Woodson *et al.*, 2011). GUN2 (heme oxygenase), GUN3 (phytochromobilin synthase), and GUN6 (FC1) operate in the heme branch of tetrapyrrole synthesis (Mochizuki *et al.*, 2001). GUN4 and GUN5 are involved in the addition of Mg to protoporphyrin IX to produce Mg–protoporphyrin IX, which is the first dedicated step in chlorophyll biosynthesis. Unlike the other GUN proteins (Vinti *et al.*, 2000; Mochizuki *et al.*, 2001), GUN1 is a chloroplast-localized pentatricopeptide repeat (PPR) protein that integrates signals from multiple sources (Koussevitzky *et al.*, 2007), although how it performs this function is unclear. Because most characterized PPR proteins are targeted to mitochondria or plastids and are involved in organellar gene expression, with known functions in RNA editing, processing, and translation (Delannoy *et al.*, 2007), GUN1 might integrate plastid signaling on the basis of a function associated with PGE.

Besides PPR proteins, proteins for the mitochondrial Transcription tERmination Factor (mTERF) family have been found to play important roles in regulating the processing of plastid transcripts (reviewed by Kleine and Leister, 2015). For example, the Arabidopsis *bsm* mutant, which is defective for an mTERF, is albinotic and displays global defects in PGE and embryo development (Babiychuk *et al.*, 2011). More specifically, BSM is required for group II intron splicing of some plastid transcripts, suggesting that defects in the processing of plastid gene transcripts can globally suppress PGE (Babiychuk *et al.*, 2011). Similarly, complete loss of other PGE regulators, such as translation initiation factor 2 (Miura *et al.*, 2007), elongation factor G (Albrecht *et al.*, 2006), and peptide release factors 1 and 2 (Meurer *et al.*, 2002; Motohashi *et al.*, 2007), results in severe suppression of PGE, leading to an albinotic or even embryo-lethal phenotype. In addition, a drastic fall in PGE can trigger PGE-dependent signaling and lead to the inhibition of photosynthesis-associated nuclear gene (PhANG) expression, even under normal growth conditions, as shown by phenotypic analysis of the Arabidopsis *prors1* mutant, which is defective for a prolyl-tRNA synthetase (Pesaresi *et al.*, 2006). However, which specific steps in PGE lead to PGE-dependent signaling is still unknown. GUN1 seems to link PGE with retrograde signaling, and contains an SMR domain found in proteins involved in DNA repair and recombination (Koussevitzky *et al.*, 2007), in addition to its PPR domain. In fact, the domain of GUN1 that contains the PPR and SMR motifs binds DNA *in vitro* (Koussevitzky *et al.*, 2007). However, the *gun1* mutation does not significantly affect plastid mRNA profiles or PGE under normal growth conditions (Woodson *et al.*, 2013), suggesting that GUN1 is either not directly involved in the regulation of PGE or that its function in PGE becomes manifest only under certain conditions. PGE signaling normally represses nuclear *Lhcb* expression in response to perturbations in chloroplast protein production. However, in *gun1* plants, expression of *Lhcb* is slightly higher than in the wild type (WT), even in the absence of overt inhibition of PGE, implying that GUN1 has a subtle effect on PhANG expression and possibly also PGE under normal growth conditions (Sun *et al.*, 2011).

In order to identify novel components of retrograde signaling, we developed an ethyl methanesulfonate (EMS) screen for mutants that displayed enhanced activity of the promoter of the *Lhcb1* gene for chlorophyll *a*/*b*-binding protein (CAB) under normal growth conditions. A series of *coe* (CAB overexpression) mutants was isolated, and the causative mutation in one of them (*coe1*) was localized to position 844 of the *AT4G02990* gene, thus demonstrating that *COE1* codes for BSM/mTERF4. Like *gun1*, *coe1* showed increased *Lhcb* mRNA expression under normal growth conditions and displayed a weak *gun* phenotype in the presence of the herbicide norflurazon (NF), which inhibits carotenoid synthesis and causes photo-oxidative damage. Defects in GUN1 or mTERF4 decreased the expression of certain plastid mRNAs in the presence of the antibiotic lincomycin (LIN) which, like spectinomycin (SPE), inhibits protein synthesis in the chloroplast. Comparative analysis revealed that in *gun1* and *coe1/mterf4*, but not in WT, *gun4*, or *gun5* plants, the processing of plastid mRNAs and expression levels of *Lhcb1* were affected in opposite ways when plants were grown in the presence of LIN or SPE. In addition, COE1 has an impact on the intracellular accumulation and distribution of GUN1, as well as on its plastid signaling activity.

## Materials and methods

### Plant materials and growth conditions

The following *A. thaliana* mutants in the Columbia (Col-1) ecotype were obtained from the Arabidopsis Biological Resource Center: *gun1* (SAIL_742_A11, a T-DNA insertion mutant; Sun *et al.*, 2011) and *gun4* (SALK_011461, a T-DNA insertion mutant). Homozygous lines were identified by PCR using gene-specific and T-DNA-specific primers (Supplementary Table S1 at *JXB* online). Mutants were backcrossed to WT plants three times before generating double mutants to segregate out additional mutations. To generate *oeCOE1/coe1* and *oeGUN1-GFP/coe1* strains, pK7FWG2-*COE1* and pB7FWG2-*GUN1* (both driven by the cauliflower mosaic virus 35S promoter), respectively, were introduced into lines homozygous for *coe1*, and *gun1* was crossed with *coe1* to obtain the double mutant *coe1 gun1*.

All mutant and WT plants were grown in climate chambers at 22 °C and 120 µmol photons m^–2^ s^–1^ on a 12h light/12h dark regime. For the NF, LIN and SPE treatments, surface-sterilized mutant and WT seeds were plated on 1/2 Murashige and Skoog (1/2 MS; Murashige and Skoog, 1962) medium (PhytoTechnology Laboratories, LLC™, USA) containing 1% sucrose and 0.8% agar supplemented with either 5 µM NF (Sandoz Pharmaceuticals, Vienna, Austria), 220 µg ml^–1^ of LIN (Sigma, St Louis, MO, USA), or 500 µg ml^–1^ of spectinomycin (Sigma).

### RNA extraction, Northern blotting, and quantitative real-time reverse transcription PCR (qRT-PCR)

RNA was extracted with a Maxwell^®^ 16 LEV simplyRNA Purification Kit (Promega, WI, USA). Northern blot analysis was performed under stringent conditions, according to Sambrook and Russell (2001). Probes complementary to nuclear or chloroplast genes were used for the hybridization experiments. Primers used to amplify the probes are listed in Supplementary Table S1. All probes used were cDNA fragments labeled with ^32^P. Signals were detected with a phosphoimager (Typhoon; GE Healthcare, Munich, Germany) and quantified using the program ImageJ. For qRT-PCR, 1 μg aliquots of total RNA, treated with DNase I (Roche Applied Science) for at least 30min, were utilized for first-strand cDNA synthesis using iScript reverse transcriptase (Bio-Rad) according to the supplier’s instructions. The qRT-PCR profiling was carried out on an iCycler Q5 real-time PCR system (Bio-Rad), using the oligonucleotide sequences listed in Supplementary Table S1. Actin was used as an internal standard. Data from three biological and three technical replicates were analyzed with Bio-Rad iQ5 software (version2.0).

### Polysome analysis

For polysome analysis, polysomes were isolated from 5-d-old seedlings according to Barkan (1993), with certain modifications. Approximately 0.3g of seedlings were frozen and ground in liquid nitrogen to a fine powder, 1ml of polysome extraction buffer [0.2M Tris/HCl (pH 9.0), 0.2M KCl, 35mM MgCl_2_, 25mM EGTA, 0.2M sucrose, 1% Triton X-100, 2% polyoxyethylene-10-tridecyl ether, 0.5mg ml^–1^ of heparin, 100mM β-mercaptoethanol, 100 µg ml^–1^ of chloramphenicol, and 25 µg ml^–1^ of cycloheximide] was added, and the tissue was ground until thawed. The samples were incubated on ice for 10min and pelleted by centrifugation for 7min at 14 000*g*. Sodium deoxycholate was added to the supernatant to a final concentration of 0.5%, after which the samples were kept on ice for 5min and then centrifuged at 12 000*g* for 15min. Next, 0.5ml samples of the supernatant were layered onto 4.4ml sucrose gradients that were prepared, centrifuged, and fractionated as described previously (Barkan, 1993). The samples were kept at 4 °C during preparation. The RNA in each fraction was isolated, separated, and transferred onto nylon membranes (Amersham Phamacia Biotech), which were probed with ^32^P-labeled probes. Signals were detected with a phosphoimager (Typhoon; GE Healthcare).

### Run-on analysis

Run-on analysis was performed according to Zoschke *et al.* (2007). Intact chloroplasts from 3g of leaves were isolated in a 40/70% Percoll step-gradient. Chloroplasts (5×10^7^) were used in *in vitro* transcription experiments, performed at 25 °C for 15min in 50mM Tris/HCl (pH 8.0), 10mM MgCl_2_, 10mM β-mercaptoethanol, 20U RNase inhibitor, and 0.2mM ATP, GTP, and CTP, in the presence of α-^32^P-UTP (10 μCi μl^–1^). Newly synthesized, labeled RNA was extracted and hybridized overnight at 42 °C to DNA fragments (1 μg) dot blotted in duplicate onto nylon membranes. The primers used for the generation of DNA probes are listed in Supplementary Table S1. Signals were detected with a phosphorimager (Typhoon; GE Healthcare) and quantified using the program ImageJ.

### Constructs for plant transformation and yeast one-hybrid assays

To generate the pK7FWG2-*COE1* and pB7FWG2-*GUN1* plasmids (in which green fluorescent protein (GFP) is fused to c-terminus of GUN1), *COE1* and *GUN1* cDNAs were amplified using the primer pair COE1-GFPs and COE1-GFPa for *COE1*, and GUN1-GFPs and GUN1-GFPa for *GUN1*, respectively (Supplementary Table S1). The PCR products were purified, and BP and LR Clonase reactions (GATEWAY Cloning; Invitrogen) were performed according to the manufacturer’s instructions to yield the final constructs pK7FWG2-*COE1* and pB7FWG2-*GUN1*.

To construct the luciferase (LUC) reporter, the promoter region of *Lhcb1.1* (–976 to –30 nt) was amplified by PCR using gene-specific primers (Supplementary Table S1). The resulting DNA fragments were digested with *Hin*dIII and *Bam*HI, and inserted into the corresponding sites in the *35S:LUC* vector (Hellens *et al.*, 2000), replacing the 35S promoter to produce PUC-*P*_*Lhcb1.1*_ *:LUC*. The vector was subsequently digested with *Hind*III and *Sac*I and inserted into the cognate sites of the binary vector pCAMBIA1301, thus generating the pCAMBIA1301- *P*_*Lhcb1.1*_ *:LUC*.

To generate pGBKT7-*GUN1* and pGADT7-*COE1*, the *GUN1* and *COE1* cDNAs were PCR amplified from Arabidopsis cDNA using PrimeSTAR^®^ HS DNA polymerase and then inserted into the *EcoR*I and *BamH*I sites of the pGBKT7 and pGADT7 vectors, respectively.

### Plant transformation

The pK7FWG2-*COE1* and pB7FWG2-*GUN1* constructs were transformed into *Agrobacterium tumefaciens* strain GV3105 via electroporation, and the resulting strains bearing the pK7FWG2-*COE1* (expressing Kan resistance *in planta*) and pB7FWG2-*GUN1* (Basta resistance) constructs were introduced into *coe1*. T1 transgenic plants were selected by screening on Basta for *GUN1* and on kanamycin for *COE1*. Homozygous transgenic plants were used in all experiments.

### Yeast two-hybrid assay

The plasmids pGBKT7-*GUN1* and pGADT7-*COE1* were co-transformed into the yeast strain AH109 using standard techniques. Growth of diploid yeast colonies on SD–His–Leu–Trp–Ade plates supplemented with 40 μg ml^–1^ of X-α-Gal would reveal a GUN1–COE1 interaction.

### Mutagenesis and mutant isolation

*P*_*Lhcb1.1*_ *:LUC* seeds were mutagenized with EMS (Redei and Koncz, 1992). F2 seeds were sterilized and planted individually in 100 x 10mm plates (150 to 200 seeds per plate) containing 1/2 MS, 1% sucrose, and 0.8% agar (pH 5.7). Five-day-old seedlings grown under light were sprayed with luciferin and immediately placed in the dark (see below) to remove the chlorophyll fluorescence, which was monitored with a CCD camera. After 5min in the dark, an LUC image was acquired with a 5min exposure to identify *coe* mutants. Putative *coe* mutants were also transferred to soil. To eliminate false positives, putative mutants were rescreened.

### LUC analysis by CCD imaging

Imaging of the activity of the firefly LUC reporter requires application of the exogenous substrate luciferin. Luciferin (Promega) was dissolved in sterile water and stored frozen in small aliquots as a 100mM stock solution. A working solution of 1mM luciferin in 0.01% Triton X-100 was applied uniformly to seedlings by spraying five times. For LUC imaging, the seedlings were kept for 5min in the dark after application of luciferin. The imaging system consisted of a high-performance CCD camera mounted in a dark chamber, a camera controller, and a computer. Image acquisition and processing were performed with the WinView software. Exposure time was 5min, unless stated otherwise.

### Positional cloning

To generate the mapping population for *COE1*, *coe1* mutant plants were crossed to WT Arabidopsis plants of the Landsberg *erecta* (L*er*) ecotype. A total of 1600 *coe1* mutant plants were selected from the segregating F2 population based on high luminescence (expression of *P*_*Lhcb1.1*_ *:LUC*) and a pale green phenotype. Genomic DNA from these plants was extracted and analyzed for co-segregation with respect to simple sequence length polymorphism (SSLP) markers. These markers were developed according to Lukowitz *et al.* (2000). Primer pairs for fine mapping of *COE1* are listed in Supplementary Table S1. In addition, nucleotide differences between L*er* and Col ecotypes were identified by direct sequencing of the ORF of *T4I9.*

### Protein extraction and immunoblot analysis

Five-day-old seedlings were harvested from 1/2 MS plates, and total proteins were prepared according to Sun *et al.* (2011). For immunoblot analyses, the proteins were fractionated by SDS-PAGE (15% acrylamide) (Schägger and von Jagow, 1987). Subsequently, proteins were transferred to polyvinylidene difluoride membranes (Ihnatowicz *et al.*, 2004) and probed with appropriate antibodies. Signals were detected by enhanced chemiluminescence (GE Healthcare).

### Thylakoid membrane preparation and blue native polyacrylamide gel electrophoresis (BN-PAGE)

Thylakoid membranes were prepared as described by Zhang *et al.* (1999). Arabidopsis leaves were ground in an ice-cold isolation buffer containing 400mM sucrose, 50mM HEPES/KOH (pH 7.8), 10mM NaCl, and 2mM MgCl_2_, filtered through two layers of cheesecloth, and centrifuged at 5000*g* for 10min. The thylakoid pellets were washed with isolation buffer, recentrifuged, and finally suspended in isolation buffer. The chlorophyll content was determined spectrophotometrically according to the method described by Porra *et al.* (1989). BN-PAGE was carried out as described previously (Schägger and Cramer, 1994). The thylakoid membranes were solubilized with 1% (w/v) dodecyl-β-maltoside in 20% glycerol, 25mM BisTris/HCl (pH 7.0), at 0.5mg chlorophyll ml^–1^ for 10min at 4 °C, and unsolubilized material was removed by centrifugation at 12 000*g* for 10min. The supernatant was combined with 0.1 vols of 5% Serva blue G in 100mM BisTris/HCl (pH 7.0), 0.5M 6-amino-*n*-caproic acid, 30% (w/v) glycerol, and loaded onto 6–12% acrylamide gradient BN gels.

### Chlorophyll fluorescence analysis

*In vivo* chlorophyll *a* fluorescence of whole seedlings was recorded using an imaging chlorophyll fluorimeter (ImagingPAM; Walz, Germany). Dark-adapted plants were exposed to a pulsed, blue measuring beam (1 Hz, intensity 4; F0) and a saturating light flash (intensity 4) to obtain Fv/Fm. A 10min exposure to actinic light (80 μmol photons m^−2^ s^−1^) was then used to calculate the steady-state magnitudes of the quantum yield of photosystem II (PSII) (ɸ_II_), non-photochemical quenching of chlorophyll fluorescence (NPQ), and the fraction of open PSII centers (qL).

### Accession numbers

Arabidopsis Genome Initiative locus identifiers for the genes mentioned in this article are *AT4G02990* (*mTERF4*/*COE1*/*BSM*/*RUG2*), *AT2G31400* (*GUN1*), *AT3G59400* (*GUN4*), and *AT5G13630* (*GUN5*).

## Results

### Identification of the *coe1* mutant

To identify components of plastid signaling under physiological conditions, we transformed Arabidopsis plants with a construct containing the *Lhcb1.1* [*LIGHT HARVESTING CHLOROPHYLL A/B-BINDING PROTEIN 1.1* or *CHLOROPHYLL A/B-BINDING PROTEIN 2* (*CAB2*)] promoter fused to the firefly LUC coding sequence. The resulting *P*_*Lhcb1.1*_ *:LUC* plants (and the *P*_*35S*_ *:LUC* transgenics used as controls) emitted luminescence under normal growth conditions (Supplementary Fig. S1 at *JXB* online). In contrast to *P*_*35S*_ *:LUC* plants, *P*_*Lhcb1.1*_ *:LUC* plants displayed significantly lower luminescence levels when grown in the presence of either LIN or NF (Supplementary Fig. S1), as expected, because both agents are known to activate plastid signaling (Dong *et al.*, 2007; Sun *et al.*, 2011; Kindgren *et al.*, 2012).

**Fig. 1.**
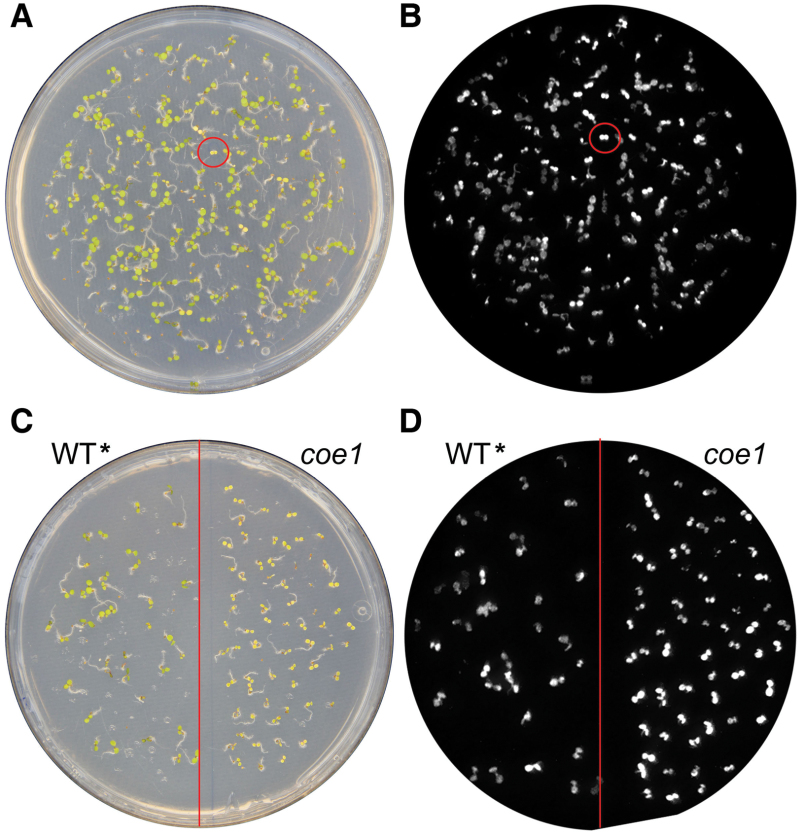
Identification of *coe* mutants. (A) Four-day-old F2 seedlings of EMS-mutagenized *P*_*Lhcb*_ *:LUC* (WT*) plants. (B) Luminescence image of the seedlings shown in (A). A putative *coe* mutant is highlighted by a circle. (C) Plate with 4-d-old WT* and *coe1* seedlings. (D) Luminescence image of the seedlings shown in (C). Note that the plate shown was the initial plate of the M2 generation from which *coe1* was isolated, and this plate was by coincidence one that contained many M2 *coe1* mutants, while on many other plates we did not identify *coe* mutants.

**Fig. 2.**
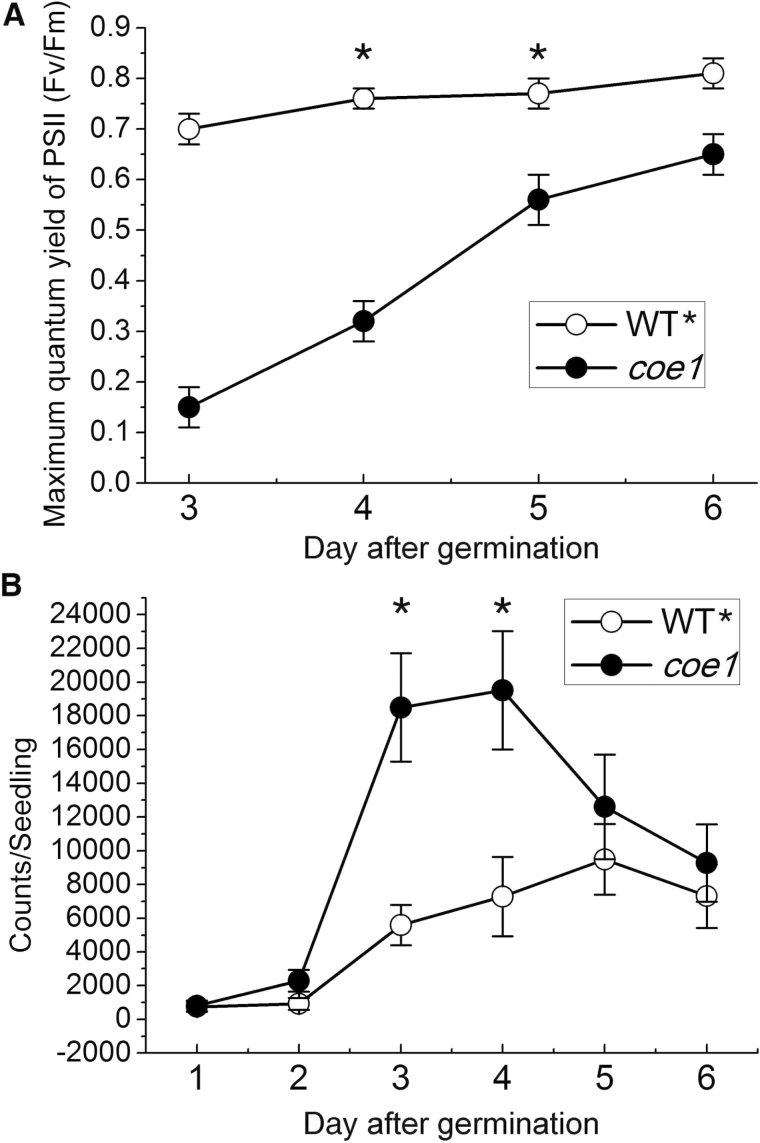
Characterization of the developmental changes in Fv/Fm and LUC activity in *coe1* and WT* seedlings. (A) Changes in Fv/Fm from day 3 to day 6 after germination. (B) Changes in LUC activity were measured from day 1 to day 6 after germination. Each data point represents the mean value for a sample of 48 seedlings. Data were expressed as mean±SD of three independent experiments. Asterisks indicate *P*<0.05 (Student’s *t*-test).

*P*_*Lhcb1.1*_ *:LUC* plants, designated in the following as WT*, were then mutagenized with EMS, and mutants with increased luminescence under normal growth conditions were identified and classified as putative *chlorophyll a/b-binding protein overexpression* (*coe*) mutants (Fig. 1A, B). We identified more than 100 *coe* mutants, and one of them, designated *coe1*, was chosen for detailed characterization. Compared with WT*, *coe1* mutants showed increased luciferase expression under normal growth conditions (Fig. 1C, D). Interestingly, *coe1* leaves were pale yellow in color, and this trait first appeared in young seedlings (Supplementary Fig. S2 at *JXB* online). As suggested by the pale yellow phenotype, the maximum quantum yield of PSII (Fv/Fm) at very early developmental stages was substantially reduced in the *coe1* strain relative to WT* but increased gradually as the seedlings got older (Fig. 2A and Supplementary Fig. S2). The higher level of luciferase expression in *coe1* was particularly evident on days 3 and 4 after germination (Fig. 2B). This phenotype suggested that COE1 may play a special role during early seedling development.

The *coe1* mutant was also backcrossed to the WT*. The resulting F1 plants all exhibited a WT* phenotype (Table 1). The F2 progeny of the selfed F1 segregated at approximately 3:1 for WT:mutant (Table 1), indicating that *coe1* is a recessive mutation in a single nuclear gene.

**Table 1.** Analysis of coe1 (WT*×coe1)^*a*^

Generation	Seedlings tested	Mutant	WT*	*P* value
F_1_	23		23	
F_2_	465	113	352	˂0.05^*b*^

^*a*^ Female×male.

^*b*^ For 3:1 segregation.

### PhANG expression in *coe1*

**Fig. 3.**
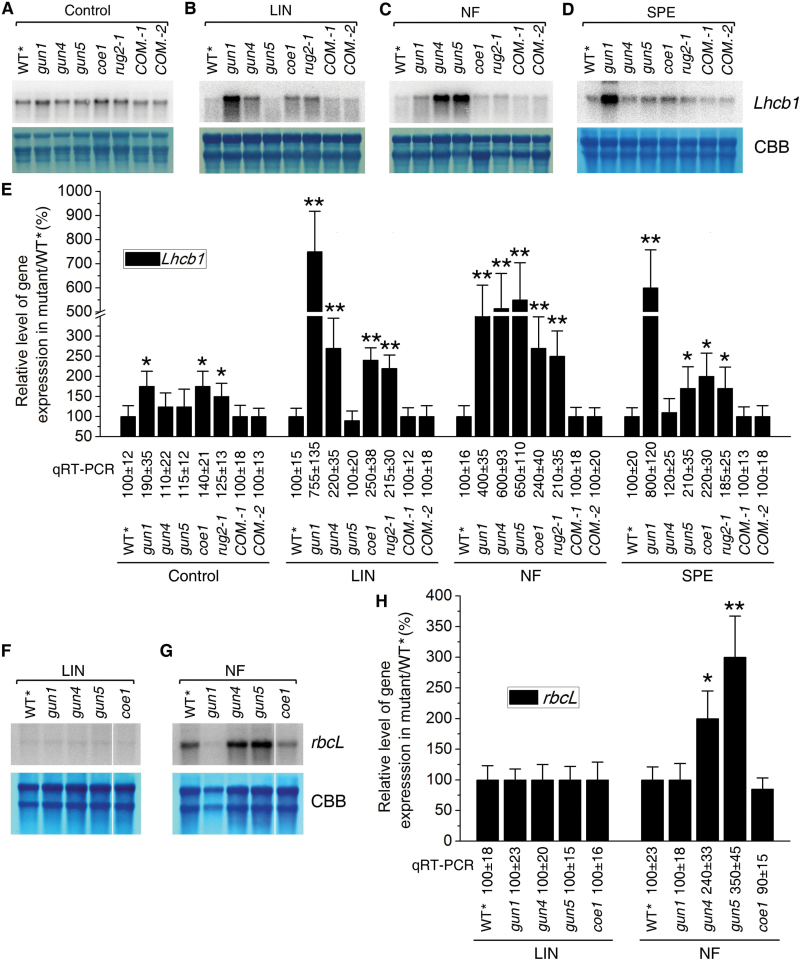
The *coe1* mutant displays a *gun* phenotype. (A–D) Levels of *Lhcb1.1* mRNA in 5-d-old WT* and mutant (*gun1*, *gun4*, *gun5*, *coe1*, and *rug2-1*) and complemented lines (*COM.-1* and *COM.-2*) seedlings grown under standard conditions (Control; A) or in the presence of LIN (B), NF (C), or SPE (D). (E, H) Signals were quantified using ImageJ software. Levels in mutants are expressed as a percentage of the WT* value. Data were expressed as means±SD of three independent experiments. *P<0.05, **P<0.01 (Student’s *t*-test versus WT*). (F, G) Levels of *rbcL* mRNA in WT* and mutant (*gun1*, *gun4*, *gun5*, and *coe1*) seedlings after treatment with LIN (F) or NF (G). The levels of *Lhcb* and *rbcL* mRNA were determined by RNA gel blots and real-time PCR analyses. The relative expression values obtained with real-time PCR analyses are indicated in the upper panel. Coomassie Brilliant Blue-stained ribosomal RNA served as the loading control (CBB).

To determine whether the *coe1* mutation also affected endogenous *Lhcb1.1* gene expression, we extracted total RNA from *coe1* and WT* seedlings grown on standard 1/2 MS plates, and performed Northern blotting and real-time PCR analyses. Fig. 3A shows that the steady-state levels of *Lhcb1.1* mRNA were higher in *coe1* than in WT* under normal growth conditions. Analysis of the changes in *Lhcb1.1* expression during development showed that enhanced *Lhcb1.1* transcript accumulation was seen at all time points between days 3 and 7 after germination, although the difference was especially evident on days 3 and 4 (Supplementary Fig. S3 at *JXB* online). In agreement with results obtained for LUC activity and Fv/Fm values, *Lhcb1.1* transcript levels in 7-d-old *coe1* were close to those of the WT* (Supplementary Fig. S3). We therefore used 3- to 5-d-old seedlings for all subsequent experiments.

To further examine the effects of the *coe1* mutation on PhANG expression, we analyzed the expression of *Lhcb1.1* in the presence of LIN, NF, or SPE (Fig. 3B–D). While after LIN treatment the expression of *Lhcb1.1* in the *gun1* and *coe1* mutants was about 750 and 250% of WT* levels, respectively, *coe1* plants treated with NF displayed almost *gun1*-like levels of *Lhcb1.1* mRNA (Fig. 3C, E). In addition, the expression of other PhANGs (*Lhcb2*, *Lhcb3*, *Lhcb4*, *RbcS1a*, and *CA1*) was also slightly higher in *coe1* than in WT* in the presence of NF (Supplementary Fig. S4 at *JXB* online), suggesting that COE1 may be involved in modulating retrograde signaling. In *gun1*, *Lhcb1.1* levels were significantly higher than in WT* in the presence of LIN, SPE, or NF. Unlike *Lhcb1.1*, accumulation of the *rbcL* transcript was strongly inhibited in all genotypes by LIN (Fig. 3F, H). Interestingly, levels of *rbcL* mRNA were significantly higher in *gun4* and *gun5* than in the other genotypes in the presence of NF (Fig. 3G, H), suggesting that *gun4* and *gun5* mitigate the effects of NF on the expression of plastid mRNAs, as has been shown for *gun5* by Ankele *et al.* (2007).

### Accumulation of photosynthetic proteins is differentially affected in *coe1*

**Fig. 4.**
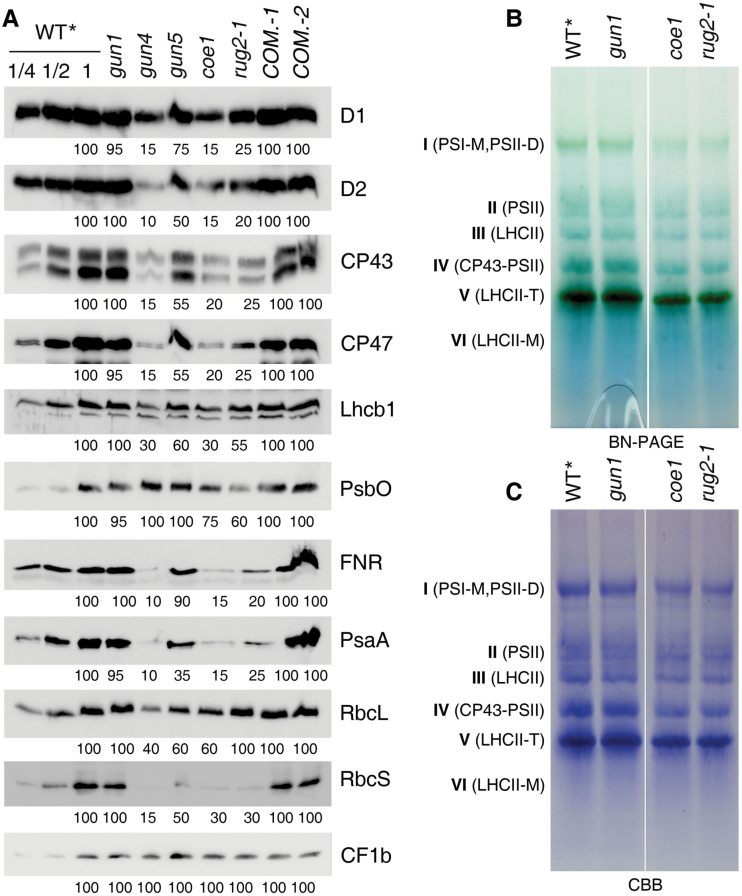
Effects of the *coe1* mutation on levels of thylakoid proteins and thylakoid complexes. (A) Selected thylakoid proteins were quantified on immunoblots. Total proteins were extracted from 5-d-old mutants (*gun1*, *gun4*, *gun5*, *coe1*, and *rug2-1*), complemented lines (*COM.-1* and *COM.-2*), and WT* seedlings, and fractionated by SDS/urea-PAGE. Blots were probed with anti-D1, -D2, -CP43, -CP47, -PsbO, -Lhcb1, -PsaA/B, -FNR, -RbcL, -RbcS, and -CF_1_β antibodies. Signal intensities (quantified by the ImageJ software), expressed relative to those of the WT* (=100), are indicated below each panel. (B) Thylakoid membranes (10 µg of chlorophyll) from WT, *gun1*, *coe1*, and *rug2-1* leaves were solubilized and separated by BN-gel electrophoresis. The positions of protein complexes representing monomeric PSI and dimeric PSII (band I: PSI-M and PSII-D), Cytb6f/ATPase/monomeric PSII (band II), light-harvesting complex II (band III: LHCII), CP43 minus PSII (band IV: CP43-PSII), trimeric LHCII (band V: LHCII-T), and monomeric LHCII (band VI: LHCII-M) are indicated. (C) The BN-gel was stained with Coomassie Brilliant Blue (CBB) to show the level of protein in each of the protein complexes.

The pale yellow leaf coloration and the photosynthetic defect (Fig. 1C, Supplementary Fig. S5 at *JXB* online) suggested that chloroplast development in *coe1* is impaired. When grown on soil, the lower photosynthetic efficiency of the *coe1* mutant resulted in a decrease in growth rate (Supplementary Fig. S6 at *JXB* online). The decrease in Fv/Fm levels observed in the *coe1* mutant during early development (Fig. 2A, Supplementary Fig. S5) might be a consequence of altered thylakoid protein levels. To address this, the steady-state levels of chloroplast proteins in 5-d-old plants was determined by immunoblot analyses with antibodies raised against representative chloroplast proteins. In fact, semi-quantitative determination of protein levels by applying a dilution series and quantification with ImageJ showed that the levels of the plastid-encoded PSI reaction center protein psaA and subunits of the PSII core subunits D1, D2, CP47, and CP43 were reduced to about 15–25% of WT* levels in *coe1* (Fig. 4A). Nucleus-encoded subunits of the oxygen-evolving complex (PsbO), light-harvesting complex II (LhcB1), and ferredoxin:NADP(H) oxidoreductase (FNR) accumulated in *coe1* to ~75%, ~30%, and ~15% of WT* levels, respectively (Fig. 4A). In contrast, the level of chloroplast ATPase protein CF_1_β was virtually equivalent to that of WT* (Fig. 4A). In some respects, *coe1* behaves like the known *gun* mutants. Like those of *coe1*, the cotyledons of the *gun4* and *gun5* mutants were pale yellow in color (Supplementary Fig. S5). Interestingly, the effect of *gun4* on the accumulation of chloroplast proteins was very similar to that of *coe1*: levels of D1, D2, CP43, CP47, PsaA, and Lhcb1 were reduced to about 10–30% of WT* levels (Fig. 4A), and amounts of FNR, RbcS1a and RbcL declined to about 10, 15, and 40% of WT* levels, respectively, in *gun4* (Fig. 4A). The *gun5* mutant showed less pronounced effects on the accumulation of the chloroplast proteins. In *gun5*, D1, D2, CP43, CP47, Lhcb1, PsbO, FNR, PsaA, RbcS1a, and RbcL accumulated to about 35–95% of WT* levels (Fig. 4A), while amounts of CF_1_β in *gun5* were almost identical to WT* (Fig. 4A). Furthermore, in the *gun1* mutant, all investigated photosynthesis proteins accumulated to or nearly to WT* levels (Fig. 4A). The significant decrease of thylakoid membrane proteins suggested that thylakoid membrane complexes are also altered in *coe1*. To investigate this, Blue Native (BN) gel analysis was performed. Indeed, the levels of thylakoid membrane complexes, such as PSI and PSII, were clearly reduced in *coe1* and *rug2-1* compared with WT* and *gun1* (Fig. 4B, C).

**Fig. 5.**
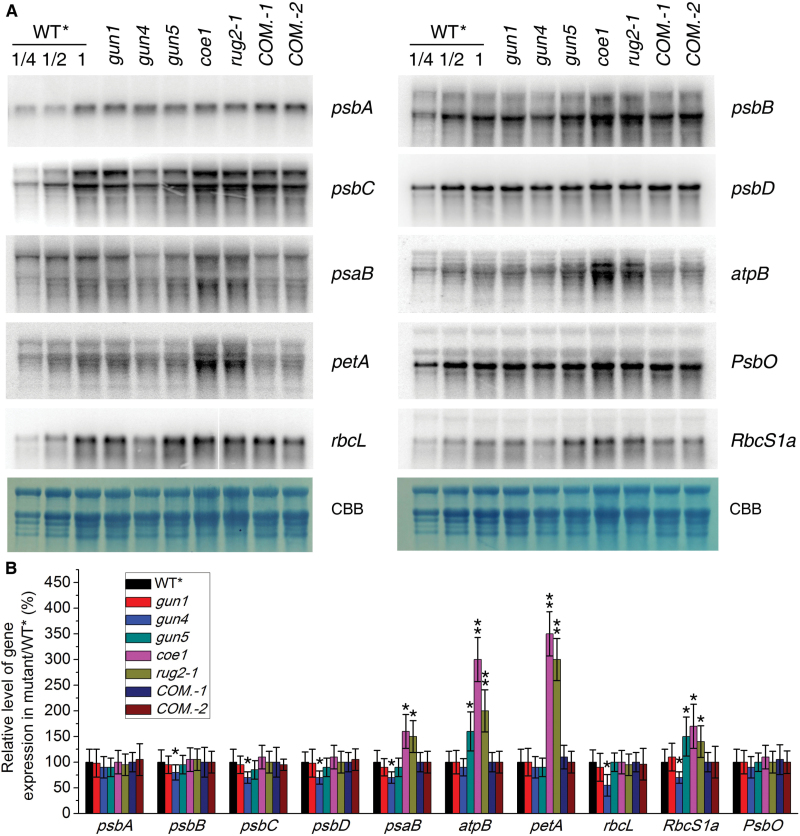
Quantification of plastid gene transcripts. (A) Aliquots (10 µg) of total RNA from WT*, mutant (*gun1*, *gun4*, *gun5*, *coe1*, and *rug2-1*), and complemented line (*COM.-1* and *COM.-2*) plants were size fractionated by agarose-gel electrophoresis, transferred to a nylon membrane, and probed with ^32^P-labeled cDNA probes derived from *psbA*, *psbB*, *psbC*, *psbD*, *atpB*, *petA*, *psaB*, *rbcL*, *RbcS1a*, and *PsbO*. Coomassie Brilliant Blue-stained ribosomal RNA served as the loading control (CBB). (B) The level of each transcript was quantified using ImageJ software, and expressed relative to that of WT*(=100%). Data were expressed as means±SD of three independent experiments. *P<0.05, ** *P*<0.01 (Student’s *t*-test versus WT*).

The dramatic reductions in chloroplast proteins observed in *coe1* could be due to decreased transcription of the corresponding genes. To assess this possibility, plastid-encoded transcripts were detected by RNA gel-blot hybridization, and levels of the transcripts were determined semi-quantitatively by applying a dilution series followed by quantification with ImageJ. This confirmed that levels of *psbA* (encoding the D1 subunit of PSII), *psbB* (encoding the CP47 subunit of PSII), *psbC* (encoding the CP43 subunit of PSII), *psbD* (encoding the D2 subunit of PSII), and *rbcL* mRNAs in the *coe1* mutant were almost identical to that of WT* (Fig. 5A, B). In contrast, levels of *atpB*, *psaB*, and *petA* transcripts (which encode the β-subunit of ATP synthase, the B subunit of PSI, and cytochrome *f*, respectively) were increased in *coe1* relative to WT* (Fig. 5A, B). In *gun4*, *psbA*, *psbB*, *psbC*, *psbD*, *psaB*, *petA*, and *rbcL* transcripts were under-represented, which was consistent with the changes in protein levels (Fig. 5A, B), whereas amounts of *atpB* and *psaB* in *gun4* were comparable to WT* (Fig. 5A, B). In *gun1* and *gun5*, levels of *psbA*, *psbB*, *psbC*, *psbD*, *rbcL*, and *psaB* RNAs were slightly decreased (Fig. 5A, B), but *atpB* and *petA* transcripts were increased relative to WT* (Fig. 5A, B). Amounts of nucleus-encoded *PsbO* mRNA in *gun1*, *gun4*, *gun5*, and *coe1* were similar to WT*, while nucleus-encoded *RbcS1a* mRNA levels were slightly increased in *gun5*, *coe1*, and *rug2-1* (Fig. 5A, B). Taken together, these results suggested that COE1 plays an important role in PGE.

Compared with WT*, the levels of chloroplast *psaB*, *petA*, and *atpB* transcripts were elevated (Fig. 5); however, the levels of the encoded proteins were reduced in *coe1* (Fig. 4), suggesting that a defect in translation of chloroplast transcripts might be responsible for reduced accumulation of the corresponding proteins. To test this, the association of *psaB*, *petA*, and *atpB* mRNA with polysomes was analyzed (Supplementary Fig. S7 at *JXB* online). To this end, plant extracts were fractionated in sucrose gradients under conditions that preserve polysome integrity, and mRNAs were identified by hybridization with specific probes. As shown in Supplementary Fig. S7, the amounts of *petA*, *psaB*, and *atpB* mRNA assembled with ribosomes (fractions 7–12) was generally similar in WT* and *coe1*, whereas mRNA accumulation of polycistronic versions of these transcripts, especially the transcript of *petA*, was higher in the non-polysomal fractions (fractions 1–6) in *coe1*. These results suggested that the majority of these mRNAs in *coe1* chloroplasts were not engaged in translation, which accounts for the reduction in synthesis of chloroplast proteins. The distribution of *petA*, *psaB*, and *atpB* mRNAs in non-polysomal and polysomal fractions of *gun1* was similar to that of WT* (Supplementary Fig. S7), suggesting that *gun1*, as expected, does not affect the translation of these proteins.

### Positional cloning of *COE1*

**Fig. 6.**
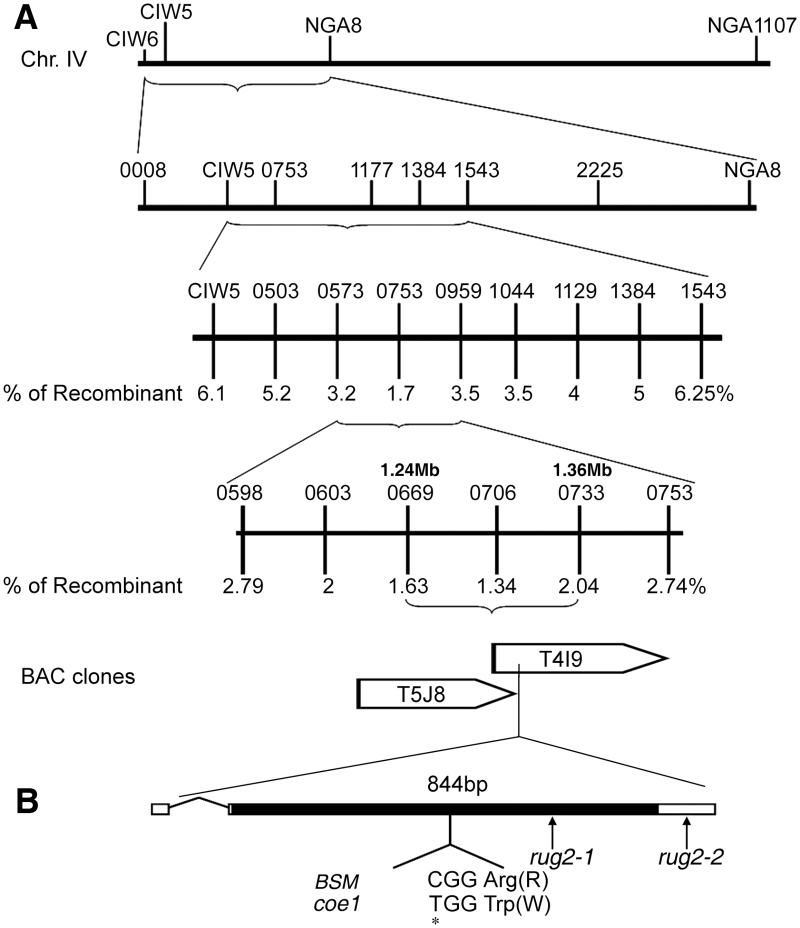
Positional cloning of the *COE1* gene. (A) Physical mapping of *COE1*. Genetic mapping delimited *COE1* to the bacterial artificial chromosome (BAC) clone T4I9. The *coe1* mutation was identified by sequencing all predicted genes on this BAC in the *coe1* mutant and comparing them with their counterparts from WT plants. (B) Structure of *COE1* and position of the *coe1* mutation. Positions are indicated relative to the initiation codon. The filled box indicates the ORF, and lines between boxes indicate introns. The nucleotide substitution in *AT4G02990* created by the *coe1* mutation is marked with an asterisk. Arrows show the T-DNA insertion positions of *rug2-1* and *rug2-2*.

To map *coe1* genetically, homozygous *coe1* mutant plants (Col ecotype) were crossed with WT plants of the L*er* ecotype. The resulting F1 plants were selfed and homozygous *coe1* mutant plants were selected from the segregating F2 population based on their pale yellow phenotype. A survey of representative molecular markers from each of the five Arabidopsis chromosomes localized *COE1* to chromosome IV (Fig. 6). Further analysis showed that *COE1* is closely linked with the SSLP marker *nga8*. Several new SSLP markers were selected from the Arabidopsis Mapping Platform (http://amp.genomics.org.cn/) and TAIR (http://www.arabidopsis.org/) between the markers *ciw6* and *nga8*. Fine-scale mapping using these new markers delimited *COE1* to the BAC clone T4I9 (Fig. 6). Candidate ORFs on T4I9 were sequenced in WT* and *coe1* mutant plants, revealing a single-nucleotide substitution in *AT4G02990* in the *coe1* mutant. This mutation was predicted to lead to a change from Arg to Trp at position 282 of the protein (Fig. 6).

To prove that the alteration in *AT4G02990* was responsible for the phenotypes observed in the *coe1* mutant, the isolated full-length *AT4G02990* cDNA was fused to the 35S promoter in the plant transformation vector pK7FWG2. The construct was introduced into *coe1* mutant plants via Agrobacterium-mediated transformation. Transformants were selected based on kanamycin resistance. Both kanamycin resistance and pale yellow leaf coloration segregated in the T2 generation. All kanamycin-resistant plants exhibited the WT* phenotype. A deeper analysis of two complemented *coe1* mutant plants in the T2 generation, *COM.-1* and *COM.-2*, demonstrated the full restoration of the WT phenotype: Luminescence of *P*_*Lhcb1.1*_ *:LUC*, Fv/Fm, ɸ_II_, and NPQ, as well as growth of COM.-1 and COM.-2 were the same as in WT plants (Supplementary Fig. S8 at *JXB* online).

### The *coe1* mutation is a new allele of *mterf4*/*bsm*/*rug2*

**Fig. 7.**
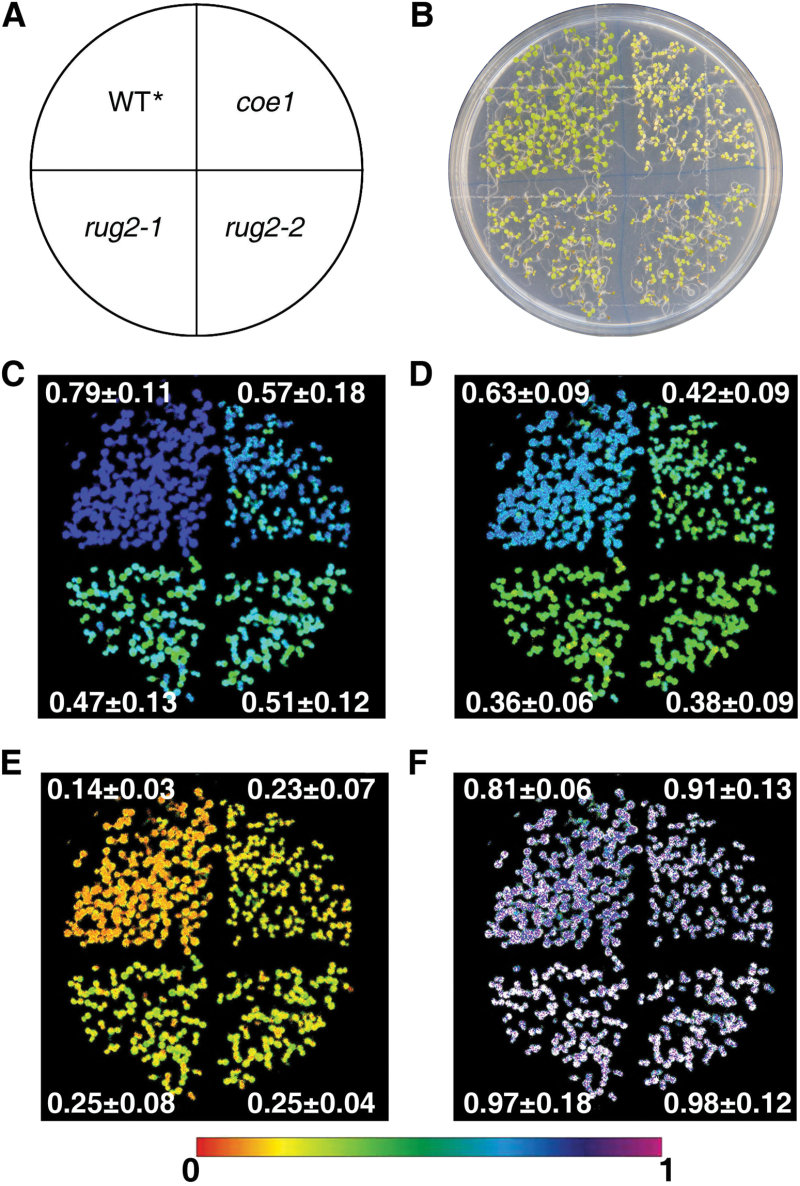
The *coe1*, *rug2-1*, and *rug2-2* mutants show similar photosynthetic defects. (A) Diagram of the plate layout showing the position of each genotype. (B) Bright-field image of *coe1*, *rug2-1*, *rug2-2*, and WT* seedlings grown on a 1/2 MS plate. (C–F) Fv/Fm (C), ɸ_II_ (D), NPQ (E), and qL (F) values for the seedlings shown in (B) were determined as described in Materials and methods. Signal intensities correspond to the color scale at the bottom of the panel. Results are shown as means±SD for Fv/Fm, ɸ_II_, NPQ, and qL in *coe1*, *rug2-1*, *rug2-2* and WT* seedlings.

Arabidopsis *AT4G02990* encodes BELAYA SMERT (BSM; Babiychuk *et al.*, 2011)/RUGOSA2 (RUG2; Quesada *et al.*, 2011), a plastid-localized mTERF protein, which has been designated mTERF4 in the systematic nomenclature of Kleine (2012). The mTERF4 protein is essential for normal plant development and for maintenance of adequate levels of transcripts in both mitochondria and chloroplasts (Babiychuk *et al.*, 2011; Quesada *et al.*, 2011). In *rug2-1*, the conserved proline residue at position 420 is replaced by leucine, and the mutant shows a variegated phenotype similar to *var1* and *var2* (Quesada *et al.*, 2011). The maize RUG2 ortholog ZmmTERF4 is localized to the chloroplast, and an allelic series of *Zmmterf4* mutants showed pale yellow/green and albino phenotypes (Hammani and Barkan, 2014). mTERF4 contains 10 mTERF motifs between aa 98 and 444, and the Arg282 to Trp mutation in COE lies in the fifth of these. In order to compare the effects of *coe1* and *rug2* on *AT4G02290*/*BSM*/*RUG2* function, we analyzed growth and photosynthesis in these genotypes (Quesada *et al.*, 2011). In fact, growth rate, cotyledon coloration, and photosynthesis parameters (Fv/Fm, ɸ_II_, NPQ, and qL) were very similar in *coe1*, *rug2-1*, and *rug2-2* (Fig. 7). In addition, as in *coe1*, the levels of chloroplast proteins D1, CP43, and CP47 in *rug2-1* were equivalent to only about 25% of WT amounts, while PsbO in *rug2-1* was reduced to about 60% of WT levels, but RbcL protein amounts were not affected in *rug2-1* (Fig. 4). Furthermore, analysis of the expression of *Lhcb1.1* revealed that *Lhcb* mRNAs were also slightly up-regulated in *rug2-1*, both under normal growth conditions and in the presence of NF and LIN (Fig. 3C, E). These results confirmed that *coe1* is a new allele of *bsm*/*rug2*.

**Fig. 8.**
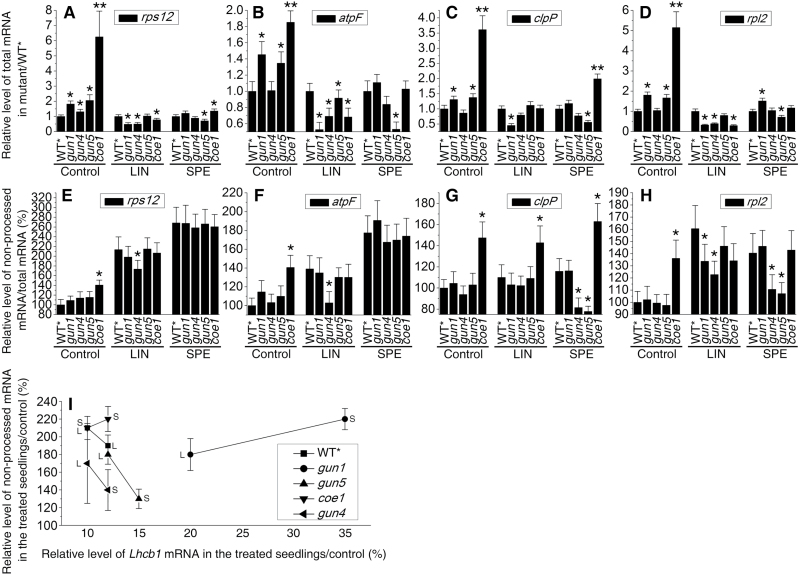
Accumulation of total and unprocessed plastid RNAs. (A–D) Relative levels of total mRNA for *rps12* (A), *atpF* (B), *clpP* (C), and *rpl2* (D) in mutants versus WT*. (E–H) Relative levels of unprocessed mRNAs for *rps12* (E), *atpF* (F), *clpP* (G) and *rpl2* (H). (I) Scatter plot for relative *Lhcb1.1* mRNA level and the relative non-processed mRNA level (means±SD) of three independent experiments for *rps12*, *atpF*, *clpP*, and *rpl2* in LIN- or SPE-treated seedlings/control. *P<0.05, ** *P*<0.01 (Student’s *t*-test versus WT*).

mTERF4 plays an important role in group II intron splicing of certain plastid transcripts, and the null *bsm* mutant seriously affected the global expression of plastid genes (Figs 5 and 8A–D) (Babiychuk *et al.*, 2011). In this respect, our *coe1* mutant showed a relatively weak *bsm* phenotype (Figs 5 and 8A–D) but, like *rug2-1*, displayed a *gun* phenotype in the presence of NF and LIN (Fig. 3). This suggested a possible connection between processing of plastid transcripts and retrograde signaling. To address this possibility, the processing of plastid-encoded transcripts was investigated in *gun1*, *gun4*, *gun5*, and *coe1* plants. As shown in Fig. 8E–H and Supplementary Fig. S9 at *JXB* online, under standard growth conditions, the processing of *atpF*, *clpP*, *rpl2*, and *rps12* was normal in *gun1*, *gun4*, and *gun5*, but was strongly perturbed in *coe1* compared with WT*. Similarly, exposure to LIN, like treatment with SPE (Babiychuk *et al.*, 2011), strongly inhibited the processing of *atpF*, *rpl2*, and *rps12* in WT* seedlings (Fig. 8E–H and Supplementary Fig. S9). Strikingly, *gun5* markedly mitigated the effects of SPE on the processing of *atpF* and *clpP* transcripts (Fig. 8E–H and Supplementary Fig. S9). In contrast, *gun1* did not alter the inhibitory effect of LIN and SPE on the processing of *atpF*, *clpP*, *rpl2*, and *rps12* RNAs (Fig. 8E–H and Supplementary Fig. S9), and further repressed the mRNA levels of *atpF*, *clpP*, *rpl2*, and *rps12* in the presence of LIN but not SPE (Fig. 8A–D and Supplementary Fig. S9). In fact, in this context, *coe1* behaved like *gun1* with respect to *atpF* and *rpl2*. In WT*, *gun4*, and *gun5* seedlings grown in the presence of LIN and SPE, levels of unprocessed chloroplast transcripts and of *Lhcb* mRNA followed opposing trends (Fig. 8I), i.e. the more unprocessed chloroplast transcripts present, the less *Lhcb1.1* mRNA was detected. However, in *gun1* and *coe1* grown with either antibiotic, disruption of plastid RNA processing was accompanied by a *rise* in levels of *Lhcb1.1* mRNA. These results suggested a link between the accumulation of non-processed transcripts and PGE-dependent signaling.

Furthermore, the higher accumulation of *atpF*, *clpP*, *rpl2*, and *rps12* in *coe1* and also partly in *gun1* (Fig. 8) and *psaB*, *atpB*, and *petA* transcripts (Fig. 5) in *coe1* could be the result of higher transcription rates of these transcripts. To test this, run-on transcription assays were carried out on isolated chloroplasts of 2-week-old WT*, *coe1*, and *gun1* seedlings. As shown in Supplementary Fig. S10 at *JXB* online, no signals were detected in the controls with the nucleus-encoded *RbcS1a* gene and the mitochondrial-encoded *atp1* gene, but the transcripts of the chloroplast-encoded genes *atpB*, *clpP*, *atpF*, *petA*, *psbC*, *psaB*, *rpl2*, and *rps12* were detected. In general, in all genotypes, *psbC*, *psaB*, and *rpl12* showed relatively strong transcription rates, while the other genes were relatively weakly transcribed (Supplementary Fig. S10). Compared with those of WT*, the transcription rate for *psaB* was 3-fold higher in *coe1*, and those for *atpF* and *clpP* were 2-fold higher in *coe1* but only slightly elevated in *gun1*, while the other transcription rates were all about the same in WT*, *coe1*, and *gun1* (Supplementary Fig. S10). These results suggested that elevated mRNA levels of some chloroplast-encoded genes in *coe1* might be caused by higher transcription rates.

### COE1 genetically interacts with GUN1

**Fig. 9.**
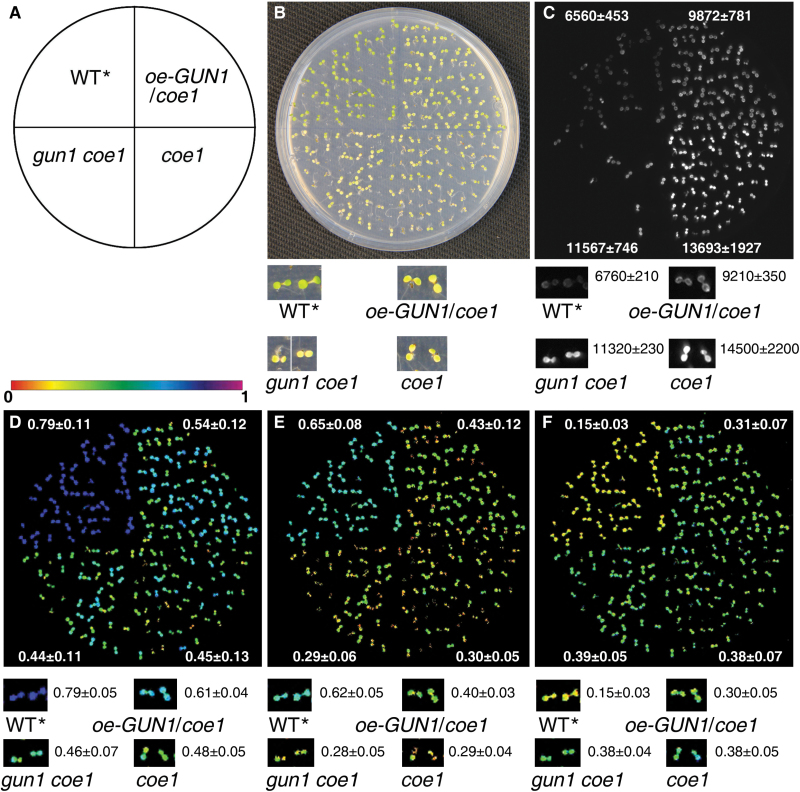
Genetic interactions between GUN1 and mTERF4. (A) Diagram of the plate layout showing the position of each genotype. (B) Bright-field images of *coe1*, *GUN1-GFP*/*coe1*, *gun1 coe1*, and WT* plants grown on a 1/2 MS plate. (C) Luminescence of the seedlings shown in (B). Note that the luminescence in some of the F2 *gun1 coe1* seedlings may be due to segregation of the *P*_*Lhcb1.1*_ *:LUC* transgene. (D–F) Fv/Fm (D), ɸ_II_ (E), and NPQ (F) of the seedlings shown in (B) was determined as described in Materials and Methods. Signal intensities for Fv/Fm are indicated according to the color scale. Results are shown as means±SD for luminescence, Fv/Fm, ɸ_II_, and NPQ.

Mutations in mTERF4 and GUN1 have similar effects on *atpF* and *rpl2* RNA processing (Fig. 8). Moreover, both proteins potentially interact with nucleic acids (Koussevitzky *et al.*, 2007; Babiychuk *et al.*, 2011, and this study). We therefore tested whether these two proteins functionally interacted with each other. To this end, we first investigated the genetic relationship between GUN1 and mTERF4 (Fig. 9). In fact, overexpression of *GUN1* (*oeGUN1-GFP/coe1*) could partially rescue the pale green phenotype of *coe1* under normal growth conditions (Fig. 9B). Levels of luminescence of *P*_*Lhcb1.1*_ *:LUC* were also slightly lower in *oe*-*GUN1-GFP*/*coe1* than in *coe1* (Fig. 9C). In parallel, the value of Fv/Fm was slightly higher in *oe*-*GUN1-GFP/coe1* than in *coe1* (Fig. 9D). These results suggested that overexpression of *GUN1* may partially compensate for the defect of *coe1* in the regulation of chloroplast biogenesis with respect to leaf coloration and Fv/Fm. In addition, we also generated a *gun1 coe1* double mutant. Compared with *coe1*, the *gun1 coe1* mutant showed a more severe leaf color phenotype and grew more slowly (Fig. 9B), but levels of *P*_*Lhcb1.1*_ *:LUC* luminescence were not affected in the *gun1 coe1* double mutant (Fig. 9C). The value of Fv/Fm of *gun1 coe1* was also lower than that in *coe1* (Fig. 9D). These results indicated that GUN1 is required to maintain chloroplast biogenesis and function when COE1 is impaired.

The potential for functional interaction between GUN1 and COE1 prompted us to test whether GUN1 could physically interact with COE1 in a yeast two-hybrid system. The coding sequence of GUN1 was cloned into the yeast bait vector pGBKT7 to generate GUN1-BD and the coding sequence of COE1 was cloned into the prey vector pGADT7 to produce COE1-AD. The resulting co-transformants expressing both GUN1-BD and COE1-AD were analyzed for growth on plates lacking His, Leu, Ade, and Trp (SD–His–Leu–Trp-Ade), and their α-galactosidase activities were assayed. As in the negative control, no interaction between GUN1 and COE1 was detectable, suggesting that the two proteins did not physically interact (Supplementary Fig. S11).

**Fig. 10.**
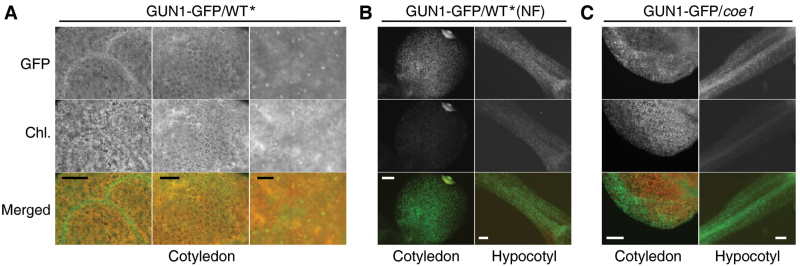
The *coe1* mutation alters the accumulation and distribution of GUN1-GFP. (A) Accumulation and distribution of GUN1–GFP in WT* under normal growth conditions. (B) Accumulation and distribution of GUN1–GFP in WT* in the presence of NF. (C) Accumulation and distribution of GUN1–GFP in *coe1* under normal growth conditions. Bars, 200 µm.

Alternatively, COE1 might affect the function of GUN1 by modulating its distribution in the plant. Analysis of the tissue localization of GUN1 fused to green fluorescent protein (GFP) indicated that the GFP fluorescence was prominent primarily in guard cells and in leaf-vein cells of cotyledons in WT* under normal growth conditions (Fig. 10A). Interestingly, in the presence of NF, enhanced GFP fluorescence was observed in all epicotyls and hypocotyls of the seedlings (Fig. 10B). In the *coe1* genetic background, the GUN1–GFP fluorescence showed a similar trend to that seen in NF-treated WT seedlings (Fig. 10C). These results suggest that *coe1* may affect the accumulation or distribution of GUN1. Alternatively, the defects in processing of plastid transcripts in *coe1* may cause a stress syndrome similar to that induced by NF, thereby changing the behavior of GUN1–GFP.

## Discussion

Plastid retrograde signaling is essential for the biogenesis and development of chloroplasts because of its impact on the expression of PhANGs (Mochizuki *et al.*, 1996; Larkin *et al.*, 2003; Koussevitzky *et al.*, 2007; Kleine *et al.*, 2009). However, in spite of its fundamental importance, little is known about the molecular nature of the process. In order to dissect the mechanism of retrograde signaling, we analyzed the expression of plastid and nuclear genes in *gun* mutants and WT seedlings. Interestingly, under normal growth conditions, levels of nucleus-encoded *Lhcb* transcripts were slightly higher in *gun* mutants than in WT at early stages of seedling development (Fig. 3A, E). Furthermore, *gun* mutants showed a subtle decrease in the expression of the plastid-encoded *rbcL* mRNA during the same developmental phase (Fig. 3F, G). These results clearly indicated that the activity of PGE is impaired in *gun* mutants. However, how the PGE-dependent signal is produced, and how it is modulated by GUN1, is unknown. In this study, we isolated a novel mutant, *coe1*, which shows up-regulation of *Lhcb1.1* expression under normal growth conditions (Figs 1 and 3A, E). The *coe1* mutant is also characterized by slightly higher PhANG expression than WT in the presence of NF and LIN (Fig. 3B, C, E and Supplementary Fig. S4), suggesting that COE1 plays a role in modulating retrograde plastid signaling.

The *coe1* mutant displayed a pale yellow leaf phenotype, suggesting that the biogenesis of chloroplasts is impaired (Supplementary Fig. S2). The maximum quantum efficiency of PSII (Fv/Fm) was indeed substantially reduced in *coe1* relative to WT and other *gun* mutants (Supplementary Figs S2 and S5). The accumulation of chloroplast proteins was also significantly decreased in *coe1* and *rug2-1* (Fig. 4), and the levels of thylakoid membrane complexes were also clearly decreased in *coe1* and *rug2-1* but almost unchanged in *gun1* compared with WT* (Fig. 4B, C). Furthermore, as a secondary effect of reduced thylakoid complex accumulation in *coe1*, Lhcb1 protein levels were also decreased (Fig. 4). Like protein levels of Lhcb1, RbcS proteins levels were also reduced in *coe1* (Fig. 4), which is likely the consequence of the reduced RbcL levels due to disturbed chloroplast translation in this mutant. Although chloroplast proteins accumulated to lower levels in *coe1*, the amount of plastid transcripts in *coe1* was about the same or even up-regulated compared with WT* (Fig. 5). This can be explained by the fact that the majority of transcripts for *petA*, *psaB*, and *atpB* might not be engaged in translation (Supplementary Fig. S7). Interestingly, in run-on experiments, levels of *psaB*, *atpF*, and *clpP* were increased in *coe1* and slightly increased in *gun1*, suggesting that at least COE1 might modulate the transcription activity of plastid genes (Supplementary Fig. S10). Unlike *prors1*, which is defective in PGE and associated with a reduction of PhANG expression under normal growth conditions (Pesaresi *et al.*, 2006), transcription of *Lhcb1.1* was increased in *coe1* (Figs 2B and 3A), while levels of Lhcb1 protein were significantly lower in *coe1* than in WT under normal growth conditions (Fig. 4).

Molecular cloning of *COE1* revealed that it codes for mTERF4, and is allelic to *BSM*/*RUG2*/*ZmmTERF4* (Babiychuk *et al.*, 2011; Quesada *et al.*, 2011; Hammani and Barkan, 2014). The mTERFs form a large and complex protein family in both metazoans and plants (Kleine, 2012; Kleine and Leister, 2015). In stark contrast to the case in mammals, the functions of mTERFs in plants are poorly understood (Kleine, 2012). The mTERF family in plants is considerably larger than in Metazoa; for example, *A. thaliana* and *Oryza sativa* Japonica contain at least 35 and 48 genes for mTERF proteins, respectively (Babiychuk *et al.*, 2011; Kleine *et al.*, 2012), all of which are predicted or known to localize to mitochondria or chloroplasts (Babiychuk *et al.*, 2011; Kleine, 2012). So far, only four plastid mTERFs—SOLDAT10, BSM, ZmmTERF4, and TWIRT1/mTERF9—have been identified and functionally characterized (Meskauskiene *et al.*, 2009; Babiychuk *et al.*, 2011; Mokry *et al.*, 2011; Quesada *et al.*, 2011). In this study, we described *coe1* as a new allele of *bsm*/*rug2/Zmmterf4* that causes similar defects in the accumulation of chloroplast proteins and the biogenesis of chloroplasts (Fig. 4). Expression of PhANGs was slightly up-regulated in *coe1* under normal growth conditions. Furthermore, *coe1* also showed a *gun* phenotype in the presence of NF (Fig. 3). Genetic analysis revealed that the effects of *coe1* on the expression of *Lhcb1* were partially dependent on GUN1 (Fig. 9). Yeast two-hybrid analysis indicated that GUN1 does not interact with COE1 (Supplementary Fig. S11), but overexpression or loss of GUN1 in *coe1* can partially rescue or aggravate its defects in the regulation of the biogenesis of chloroplasts. More interestingly, *coe1* can also regulate GUN1 function by affecting its accumulation and distribution. For instance, compared with WT, both the level and the distribution of GUN1–GFP fluorescence are altered in *coe1* under normal growth conditions (Fig. 10). Taken together, these results suggest that GUN1 and COE1 do interact at some level in regulating the expression of plastid genes and PhANGs under certain physiological conditions.

In WT*, LIN and SPE treatments lead to the accumulation of non-processed RNA (Fig. 8). The *coe1* mutant accumulates high levels of unprocessed RNAs even under normal growth conditions (Fig. 8). The *gun1* mutation did not dramatically alter RNA processing in the presence of LIN but instead seemed to affect the expression of plastid genes (Fig. 8). Alterations in chlorophyll metabolism might affect the processing of transcript stability/maturation, because for example, the chlorophyll-deficient mutants *atcrs1* and *atcaf2* (Asakura and Barkan, 2006) and rice white stripe leaf (*wst*) (Tan *et al.*, 2014) show defects in the processing of plastid transcripts. The *gun4* and *gun5* mutants, in which chlorophyll metabolism is perturbed, indeed showed subtle alterations in the processing of *rps12* and *atpF* transcripts under normal conditions but not in the presence of LIN and SPE (Fig. 8). Comparative analysis revealed that levels of unprocessed plastid transcripts are negatively correlated with expression levels of *Lhcb1.1* in WT plants exposed to LIN and SPE, and in *gun4* and *gun5* plants, but not in *gun1* or *coe1*. These results suggest that the accumulation of unprocessed plastid transcripts might trigger plastid signaling to inhibit gene expression of nuclear photosynthesis genes. In addition, altered mTERF4 levels affected the intracellular accumulation and distribution of GUN1, as well as its plastid signaling activity. Taken together, these results suggest that GUN1 and COE1 cooperate in PGE and retrograde signaling (Supplementary Fig. S12).

## Supplementary data

Supplementary data are available at *JXB* online.

**Table S1.** List of oligonucleotides used in this study.

**Fig. S1.** LUC activity in *P*_*Lhcb1.1*_ *:LUC* plants can be suppressed by treatments with LIN or NF.

**Fig. S2.** The *coe1* phenotype is especially prominent during early development of chloroplasts.

**Fig. S3.** Quantification of steady-state *Lhcb1.1* mRNA levels in *coe1* and WT* plants during early plant development.

**Fig. S4.** Transcripts of PhANGs are slightly increased in NF-treated *coe1* plants.

**Fig. S5.** Photosynthetic performance of mutant (*gun1*, *gun4*, *gun5*, *coe1*, *rug2-1* and *rug2-2*) and WT* plants.

**Fig. S6.** Growth phenotype of *gun1*, *gun4*, *gun5*, *coe1* and WT* on soil. Plants were grown on soil in a climate chamber for 3 weeks, on a 12-h light/12-h dark regime.

**Fig. S7.** Polysome association analysis for chloroplast transcripts in WT*, *coe1* and *gun1* plants.

**Fig. S8.** Complementation of the *coe1* mutation by *AT4G02990*.

**Fig. S9.** Analysis of plastid transcript processing in *gun1*, *gun4*, *gun5*, *coe1*, and WT*.

**Fig. S10.** Transcription rates of plastid genes in WT*, *coe1* and *gun1* seedlings.

**Fig. S11.** GUN1 does not interact with COE1 in yeast-two-hybrid experiments.

**Fig. S12.** A model for the functional relationship of mTERF4 and GUN1.

Supplementary Data

## Abbreviations:

BAC: bacterial artificial chromosome
BN: blue native
EMS: ethyl methanesulfonate
GFP: green fluorescent protein
LIN: lincomycin
LUC: luciferase
mTERF: , mitochondrial transcription termination factor
NF: norflurazon
PGE: plastid gene expression
PhANG: photosynthesis-associated nuclear gene
PPR: pentatricopeptide repeat
PS: photosystem
qRT-PCR: quantitative real-time reverse transcription PCR
SPE: spectinomycin
SSLP: simple sequence length polymorphism
WT: wild type.
