# Supplementary material for: Functional relationship between mTERF4 and GUN1 in retrograde signaling
Source: J Exp Bot. 2015 Dec 18;67(13):3909–24. doi: 10.1093/jxb/erv525 (PMC4915522; doi:10.1093/jxb/erv525)
Supplement: Supplementary Data [file supp_67_13_3909__index.html]

Functional relationship between mTERF4 and GUN1 in retrograde signaling — Supplementary Data 

# Functional relationship between mTERF4 and GUN1 in retrograde signaling

## Supplementary Data

Data files

- supplementary\_table\_S1\_figures\_S1\_S12.pdf - Supplementary Data
