# Supplementary material for: Functional relationship between mTERF4 and GUN1 in retrograde signaling
Source: J Exp Bot. 2015 Dec 18;67(13):3909–24. doi: 10.1093/jxb/erv525 (PMC4915522; doi:10.1093/jxb/erv525)
Supplement: Supplementary Data [file supp_erv525_supplementary_table_S1_figures_S1_S12.pdf]

Supplementary Data

Supplementary Table S1. List of oligonucleotides used in this study.

Gene	Name	Sequence (5' ------> 3')
**Plant transformation**		
COE1	COE1s	AAAAAGCAGGCTTCATGAAGATTAGGTTCTGT
	COE1a	AGAAAGCTGGGTGTGCAAACTCCTCGTCGTC
GUN1	GUN1-GFPs	AAAAAGCAGGCT TCCTTTCAATGGCGTCAACG
	GUN1-GFPa	AGAAAGCTGGGTACAAAAGAAGAGGCTGTAAAGCAA ACG
Lchb1	LHCB1.1-LUCs	CCCAAGCTTAGTTTAGAATGTGCTCTAAAGC
	LHCB1.1-LUCa	CGCGGATCCTGACTAACTTGTGAGTGAGAGTG
	adapter AttB1	GGGGACAAGTTTGTACAAAAAAGCAGGCT
	adapter AttB2	GGGGACCACTTTGTACAAGAAAGCTGGGT
**Mapping position assay**		
	CIW12s	AGGTTTTATTGCTTTTCACA
	CIW12a	CTTTCAAAAGCACATCACA
	CIW1s	ACATTTTCTCAATCCTTACTC
	CIW1a	GAGAGCTTCTTTATTTGTGAT
	ATPASEs	GTTCACAGAGAGACTCATAAACCA
	ATPASEa	CTGGGAACGGTTCGATTCGAGC
	CIW2s	CCCAAAAGTTAATTATACTGT
	CIW2a	CCGGGTTAATAATAAATGT
	CIW3s	GAAACTCAATGAAATCCACTT
	CIW3a	TGAACTTGTTGTGAGCTTTGA
	CIW11s	CCCCGAGTTGAGGTATT
	CIW11a	GAAGAAATTCCTAAAGCATTC
	CIW4s	GTTCATTAAACTTGCGTGTGT
	CIW4a	TACGGTCAGATTGAGTGATTC
	CIW5s	GGTTAAAAATTAGGGTTACGA
	CIW5a	AGATTTACGTGGAAGCAAT
	CIW6s	CTCGTAGTGCACTTTCATCA
	CIW6a	CACATGGTTAGGGAAACAATA
	CIW8s	TAGTGAAACCTTTCTCAGAT
	CIW8a	TTATGTTTTCTTCAATCAGTT
	CIW10s	CCACATTTTCCTTCTTTCATA
	CIW10a	CAACATTTAGCAAATCAACTT
	CIW9s	CAGACGTATCAAATGACAAATG
	CIW9a	GACTACTGCTCAAACTATTCGG
	NGA280s	GGCTCCATAAAAAGTGCACC

NGA280a	CTGATCTCACGGACAATAGTGC
NGA172s	CATCCGAATGCCATTGTTC
NGA172a	AGCTGCTTCCTTATAGCGTCC
NGA162s	CTCTGTCACTCTTTTCCTCTGG
NGA162a	CATGCAATTTGCATCTGAGG
NGA6s	ATGGAGAAGCTTACACTGATC
NGA6a	TGGATTTCTTCCTCTCTTCAC
NGA8s	TGGCTTTCGTTTATAAACATCC
NGA8a	GAGGGCAAATCTTTATTTCGG
NGA225s	TCTCCCCACTAGTTTTGTGTCC
NGA225a	GAAATCCAAATCCCAGAGAGG
NGA151s	CAGTCTAAAAGCGAGAGTATGATG
NGA151a	GTTTTGGGAAGTTTTGCTGG
NGA1107s	CGACGAATCGACAGAATTAGG
NGA1107a	GCGAAAAAACAAAAAAATCCA
NGA76s	AGGCATGGGAGACATTTACG
NGA76a	GGAGAAAATGTCACTCTCCACC
NGA361s	ACATATCAATATATTAAAGTAGC
NGA361a	AAAGAGATGAGAATTTGGAC
NGA1126s	GCACAGTCCAAGTCACAACC
NGA1126a	CGCTACGCTTTTCGGTAAAG
NGA168s	GAGGACATGTATAGGAGCCTCG
NGA168a	TCGTCTACTGCACTGCCG
FW1M12s	GGCTTTCTCGAAATCTGTCC
FW1M12a	TTACTTTTTGCCTCTTGTCATTG
4-AC002330-0598s	TAATGGCATAATTTACATTT
4-AC002330-0598a	TGACGAAAGTACCCTTGTAT
4-AC002330-0630s	AACCATCCTTTGTTTGATAA
4-AC002330-0630a	GCATTTGAGATTTGGAAGTT
4-AF069442-0706s	TTATAGCAAACGTACAAGTC
4-AF069442-0706a	CTGCATACACGTCGTCTC
4-AF069442-0733s	GCATTAGCGTGCAATTAAGC
4-AF069442-0733a	ATCAACCCCAAAAATGCATA
4-AC004044-0669s	CCGGAGAATCGTACGGTAC
4-AC004044-0669a	AAGCTTCGAACTCAAGGTTC
4-AF058919-0150s	AGTTCCTTCCATGCCTTAAT
4-AF058919-0150a	CAATCAAATGGAGCCAAATA
4-AF007269-0295s	GTCGGACCACAGTTGATAAG
4-AF007269-0295a	ACGGCCCGATTTGTTTAC
4-AF001308-0503s	CGATTCCTCCACCCTAATAA
4-AF001308-0503a	AATCCAACGAGAGCGAATTT
4-AF069298-0573s	ACTATCCTTGTGCAGATATT

	4-AF069298-0573a	CAAAAGAGCAAAAGCATATT
	3-AC009177-0656s	TCGATTTTGTTTTACTATCT
	3-AC009177-0656a	AATTAAGGAATCAATTACAC
	4-AF077408-0959s	TTTCACTTGATTTTGATTAT
	4-AF077408-0959a	CAGAAACTTCGCGTGTAG
	4-AF075598-1044s	AGCCATGGTTGTTGTGTCTG
	4-AF075598-1044a	AGAGGTCGTGGACGTGGA
	4-AF069441-1129s	GACACGGATTTGGAAACTTA
	4-AF069441-1129a	GTTGGTTCCGGATGTGTC
	4-AF162444-1384s	CTGAATCTCCCAGTTTATTT
	4-AF162444-1384a	AAGATCCAGGACATATAAGC
	4-AC005275-0753s	TTCAACCAGAGATTCAATAC
	4-AC005275-0753a	TGCTTCCATCATTTTGTA
	4-AF069299-0008s	GTGTCATCCTTGAGTTTATT
	4-AF069299-0008a	TTAGTGTTGGCCTTTTAAGT
	4-AF076243-1177s	TATGTGTGGAAGTTGTGTAA
	4-AF076243-1177a	TCTTCGTCATCATAAGAAAT
	4-AF069299-0008s	GTGTCATCCTTGAGTTTATT
	4-AF069299-0008a	TTAGTGTTGGCCTTTTAAGT
	4-AF147260-2225s	CTCCGATGAATCACCATTAG
	4-AF147260-2225a	CCGGTTTGGCTTGTCTGTTC
	4-AF147263-1543s	TTGAAAATTGAAACTGAATAG
	4-AF147263-1543a	AAAATCCCCAATTTGGATA
	4-AF162444-1384s	CTGAATCTCCCAGTTTATTT
	4-AF162444-1384a	AAGATCCAGGACATATAAGC
**Yeast two-hybrid assay**		
COE1	COE1-ADs	CCGGAATTCATGAAGATTAGGTTCTGTAATG
	COE1-ADa	CGCGGATCCTGCAAACTCCTCGTCGTCATC
GUN1	GUN1-BDs	GTGAATTCGCTCATCTTTCACAGACTACTC
	GUN1-BDa	GTGGATCCCACAGAGCCAAACATTGTTAGG
**Mutant confirmed**		
	GUN1LP	ATGTTTAGTAGCCACGCATGG
	GUN1RP	TTGATCGATGGGTACTCGAAG
	GUN4LP	GCCCATGTGCCTAGAAGCTAG
	GUN4RP	TGGAGAACCATCTCGTCAATC
	SalkLB	ATTTTGCCGATTTCGGAAC
	SailLB	TAGCATCTGAATTTCATAACCAATCTCGATAC
**Real-time RT-PCR**		
LHCB1	LHCB1s	ATGGCCGCCTCAACAATGG
	LHCB1a	CGGTAAGGTAGCTGGGTGAC
LHCB2	LHCB2s	ATGGCCACATCAGCTATCC

	LHCB2a	CTCCAGTTAAGTAAGACGGTGTG
LHCB3	LHCB3s	AATGATCTTTGGTATGGACCTGAC
	LHCB3a	CCACACGGACCCACTTTTG
LHCB4	LHCB4s	CAGCCGTACACTGAAGTCTTTGG
	LHCB4a	TTCTATCCATATCAACGTCGTCAAC
CA1	CA1s	TGGGAACCGAAGCATACGAC
	CA1a	ACAAAGCAGGGTTGGTTTCG
RbcS	RbCS1As	CTACTATGGTTGCCTCTCCGG
	RbCS1Aa	CAATTCGGAATCGGTAAGGTC
FNR	FNRs	ATGGCGACTACCATGAATGC
	FNRa	ACTTTCTTTGCAGGAGTAGGAG
actin	Actrt1s	GGTAACATTGTGCTCAGTGGTG
	Actrt1a	CTCGGCCTTGGAGATCCACATC
**Northern blotting/Run-on/polysomal association**		
atpF	ATPFs	ATGAAAAATTTAACCGATTCTTTC
	ATPFa	TTAATCAGTTATTTCTTTCATCGTAC
clpP	CLPPs	CCTATTGGCGTTCCAAAAGTAC
	CLPPa	ATTGAACCGCTACAAGATCAAC
rpl2	RPL2s	ATGGCGATACATTTATACAAAAC
	RPL2a	CTATTTACTACGGCGACGAAG
rps12	RPS12s	TCACCCCCAAAAAACCAAAC
	RPS12a	TATTTTGGCTTTTTGACCCC
psbA	PSBAs	TTATCCATTTGTAGATGGAGCCTCA
	PSBAa	ATGACTGCAATTTTAGAGAGACGCG
psbB	PSBBs	GCAAGGATCCATGGGTTTGCCTTGG
	PSBBa	GCAACTCGAGATCAGACTGCTTGTCG
psbC	PSBCs	GCGGGATCCATGAAAACCTTATATTCC
	PSBCa	GGCCTCGAGTTAGTTAAGAGGAGTCATG
psbD	PSBDs	TGTTCGGAAATGGTTGAAGTAGATG
	PSBDa	GGTAGAACCTCCTCAGGGAATATAA
psaB	PSABs	GTATTGCTACCGCACATGAC
	PSABa	CCACGAAACTCTTGGTTTCC
petA	PETAs	TATGATTGGTTCGAAGAACGTCTTG
	PETAa	TTATAAGGGACCAGAAATACCTTGC
PsbO	PSBO2s	AGACGGAAGCGTGAAGTTCA
	PSBO2a	CAATCTGACCGTACCAAACC
rbcL	RBCLs	CGTTGGAGAGACCGTTTCTT
	RBCLa	CAAAGCCCAAAGTTGACTCC
Lhcb1	LHCB1s	GACTTTCAGCTGATCCCGAG
	LHCB1a	CGGTCCCTTACCAGTGACAA

Supplementary Figure legends

Supplementary Figure S1. LUC activity in $P_{Lhcb1.1}$:*LUC* plants can be suppressed by treatments with LIN or NF. Bright-field image of $P_{Lhcb1.1}$:*LUC* and P_{35S}:*LUC* plants grown on 1/2 MS plates (Control) (A), and 1/2 MS plates containing NF (B) or LIN (C), respectively. (D-F) Luminescence images of the plants shown in (A-C).

Supplementary Figure S2. The *coe1* phenotype is especially prominent during early development of chloroplasts.

Seeds of *coe1* and WT***** were directly germinated on 1/2 MS plates supplemented with 1% sugar. Bright-field images of the plants are shown in the upper panel. Fv/Fm was measured with an imaging PAM as described in Materials and Methods, and signal intensities are indicated according to the color scale at the bottom.

Supplementary Figure S3. Quantification of steady-state *Lhcb1.1* mRNA levels in *coe1* and WT* plants during early plant development. Results are the averages of three independent experiments. Error bars represent standard deviations. Data were expressed as means (±SD) of three independent experiments. **p*<0.05, Student's t-test versus WT*.

Supplementary Figure S4. Transcripts of PhANGs are slightly increased in NF-treated *coe1* plants. Total mRNA was extracted from 5-day-old, NF-treated *coe1* and WT***** seedlings. After cDNA synthesis, qRT-PCR analyses were performed with primers specific for *Lhcb1*, *Lhcb2*, *Lhcb3*, *Lhcb4*, *CA1*, *RbcS1a* and *FNR*. Data are given as means (±SD) of three independent experiments. **p*<0.05, Student's t-test versus WT*.

Supplementary Figure S5. Photosynthetic performance of mutant (*gun1, gun4, gun5, coe1, rug2-1* and *rug2-2*) and WT* plants.

(A) Bright-field images of mutant (*coe1*, *gun1*, *gun4*, *gun5*, *rug2-1* and *rug2-2*) and WT***** seedlings grown on 1/2 MS plates. (B) Fv/Fm values for the seedlings shown in (A) were measured with an imaging PAM as described in Materials and Methods, and signal intensities (mean Fv/Fm value ±SD) are indicated according to the color scale at the bottom of the panel.

Supplementary Figure S6. Growth phenotype of *gun1, gun4, gun5, coe1* and WT* on soil. Plants were grown on soil in a climate chamber for 3 weeks, on a 12-h light/12-h dark regime.

Supplementary Figure S7. Polysome association analysis for chloroplast transcripts in WT*, *coe1* and *gun1* plants. Shown is the association of *petA*, *psaB*, and *atpB* transcripts with polysomes. Total extracts from WT* and *coe1* and *gun1* leaves grown on 1/2 MS plates for 5 days at 120 µmol photons m^{-2} s^{-1} were fractionated on 15%-to-55% sucrose gradients. Twelve fractions of equal volume were collected from the top to the bottom of the sucrose gradients, and equal proportions of the RNA purified from each fraction were analyzed by gel-blot analysis. Coomassie brilliant blue (CBB)-stained ribosomal RNA served as a loading control.

Supplementary Figure S8. Complementation of the *coe1* mutation by *AT4G02990.* (A) Shown are four-day-old WT***** and *coe1* seedlings, and two complemented lines (designated *COM.-1* and *COM.-2*) grown on a 1/2 MS plate (B). (C) Luminescence of the seedlings shown in (B). (D) Fv/Fm, ϕ_{II} (E), NPQ (F) of the seedlings shown in (B) were determined as described in Materials and Methods. Signal intensities are indicated according to the color scale to the right of the panel. Mean values (±SD) for luminescence, Fv/Fm, ϕ_{II}, and NPQ are shown.

Supplementary Figure S9. Analysis of plastid transcript processing in *gun1, gun4, gun5, coe1,* and WT*.

Processing of plastid transcripts in WT*****, *gun1*, *gun4*, *gun5*, and *coe1* mutants under normal growth conditions (Control) (A), or after treatment with LIN (B) or SPE (C). RNA was isolated and the levels of processing intermediates and mature transcripts of *rpl2*, *rps12*, *atpF* and *clpP* RNAs were determined by gel-blot analysis. Coomassie brilliant blue-stained ribosomal RNA served as a loading control (CBB). Examples of processed (S) and unprocessed (U) and unprocessed group II intron (U^{G2}) transcripts are marked. Quantification of signals is shown in Figure 8.

Supplementary Figure S10. Transcription rates of plastid genes in WT*, *coe1* and *gun1*

seedlings. (A) Dot blot analysis of run-on RNA samples derived from plastids isolated from leaves of 2-week-old WT*, *coe1* and *gun1* seedlings grown on 1/2 MS plates. RNA was hybridized to DNA fragments representing plastid *atpB*, *clpP*, *atpF*, *petA*, *psbC*, *psaB*, *rpl2* and *rps12* genes. The *RbcS1a* and *atp1* DNA fragments were used as controls for a possible contamination with nuclei or mitochondria, respectively. (B) Relative rates of transcription in WT* (=1), *coe1* and *gun1* leaves for *atpB*, *clpP*, *atpF*, *petA*, *psbC*, *psaB*, *rpl2* and *rps12* as determined in (A). The level of each blot was quantified by the ImageJ software, data are given as means (±SD) of three independent experiments. *$p<0.05$, ** $p<0.01$, Student's t-test versus WT*.

Supplementary Figure S11. GUN1 does not interact with COE1 in yeast-two-hybrid experiments.

Cells that had been cotransformed with the prey vector COE1-AD (mature full-length COE1 fused to the activation domain of Gal4) and the bait vector GUN1-BD (mature full-length GUN1 fused to the binding domain of Gal4) could not grow on SD/-Leu-Trp-His-Ade/X-α-Gal plates. Yeast cells cotransformed with pGADT7-T (which encodes the Gal4 AD fused to SV40 large T-antigen) and pGBKT7-53 (which encodes the Gal4 DNA-BD fused to murine p53) were used as positive (P) controls; constructs cotransformed with pGADT7-T and pGBKT7-Lam (which encodes the Gal4 BD fused to lamin) were used as negative (N) controls.

Supplementary Figure S12. A model for the functional relationship of mTERF4 and GUN1.

The mTERF4 protein is required for the processing of pre-mRNA and inhibits the accumulation of GUN1. GUN1 may be involved in the regulation of DNA repair, mRNA processing or translation. mTERF4 might modulate retrograde signaling either via GUN1 or by virtue of its effects on PGE.

Supplementary Figures

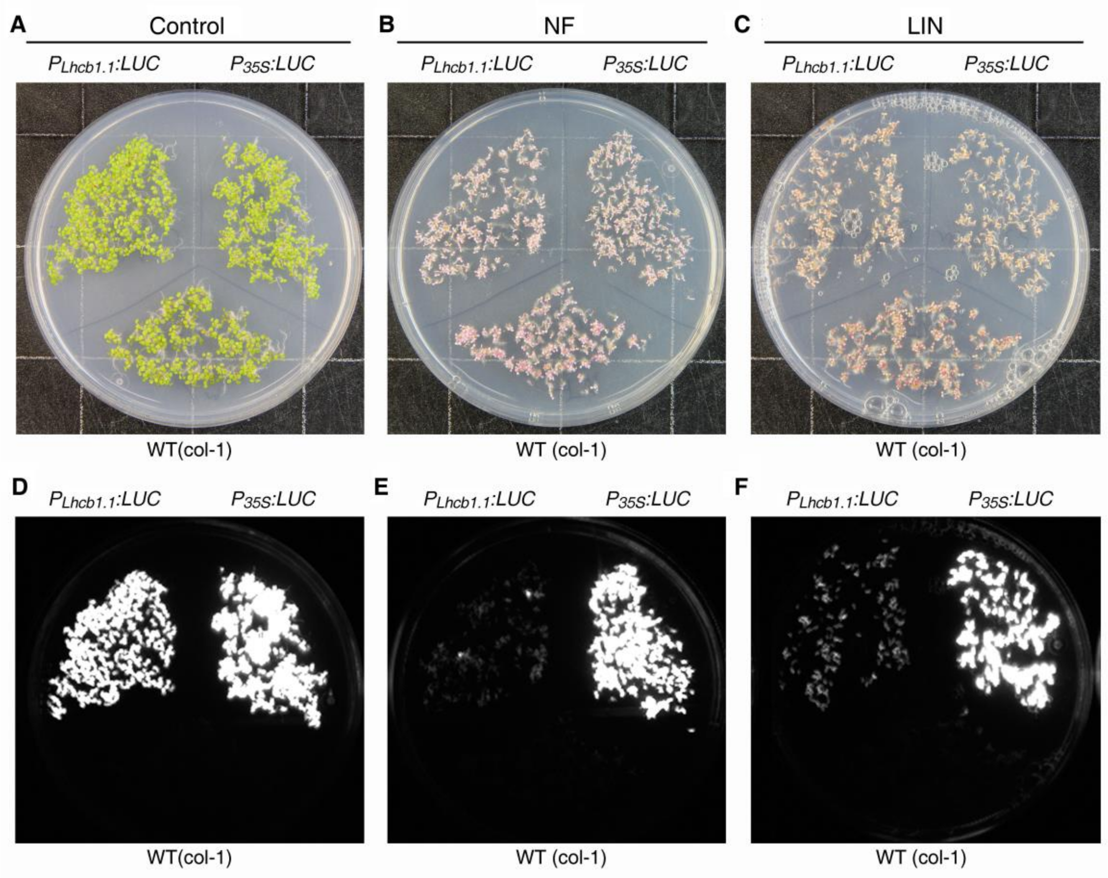

Figure S.1

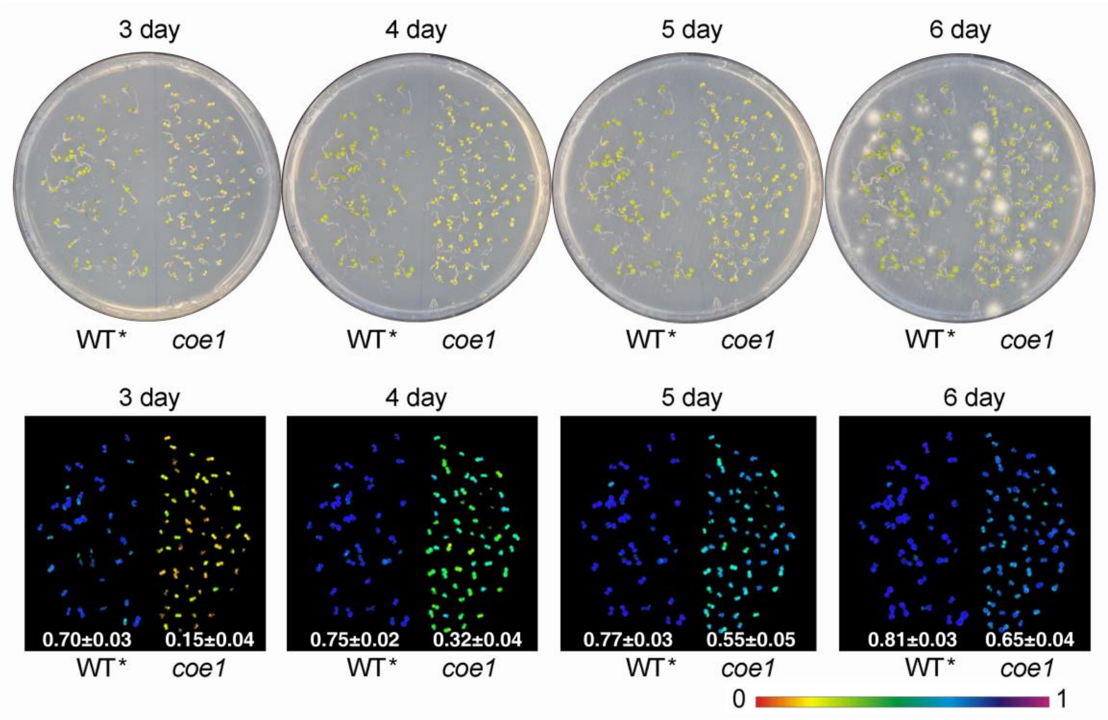

Figure S.2

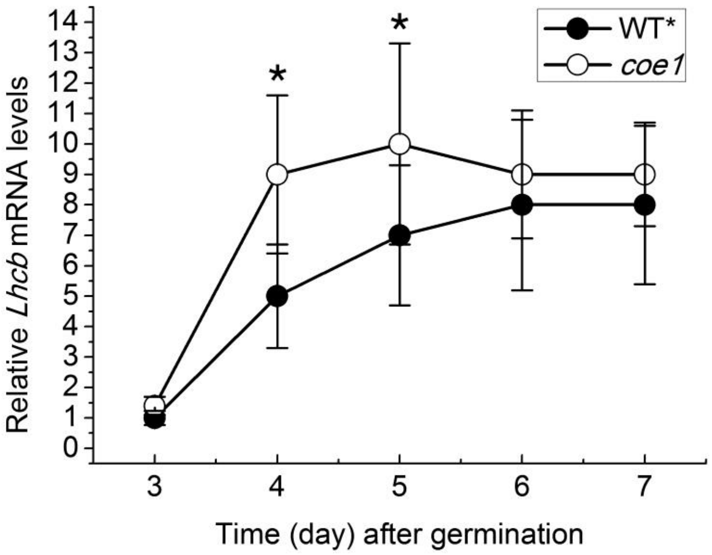

Figure S.3

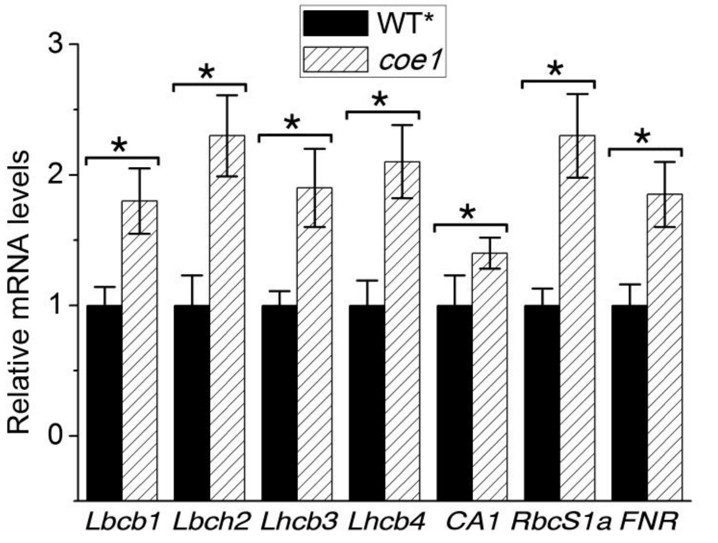

Figure S.4

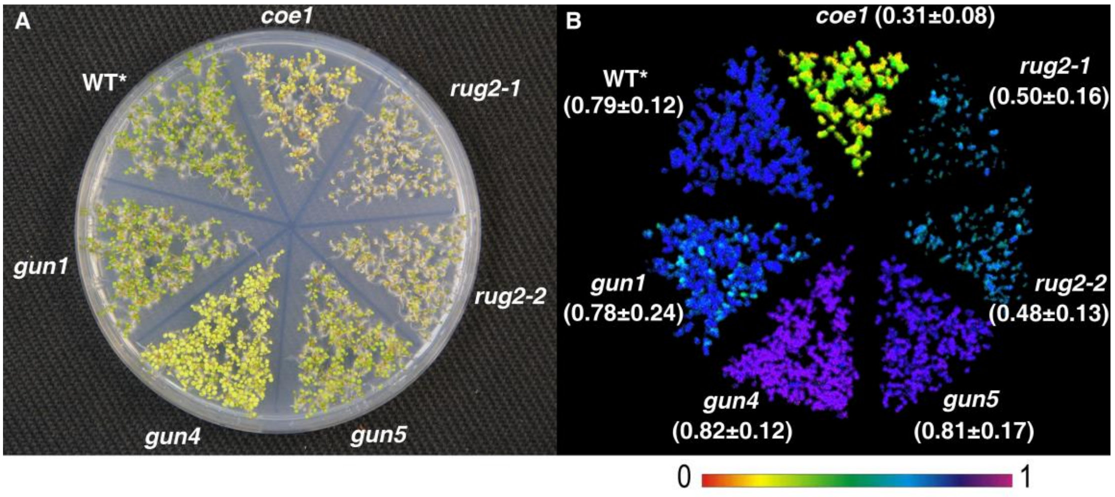

Figure S.5

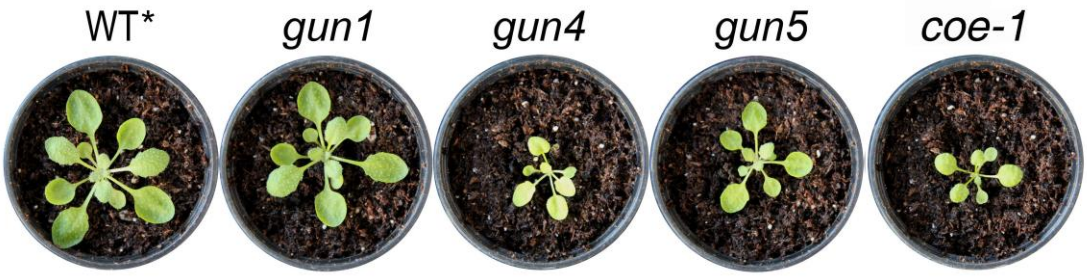

Figure S.6

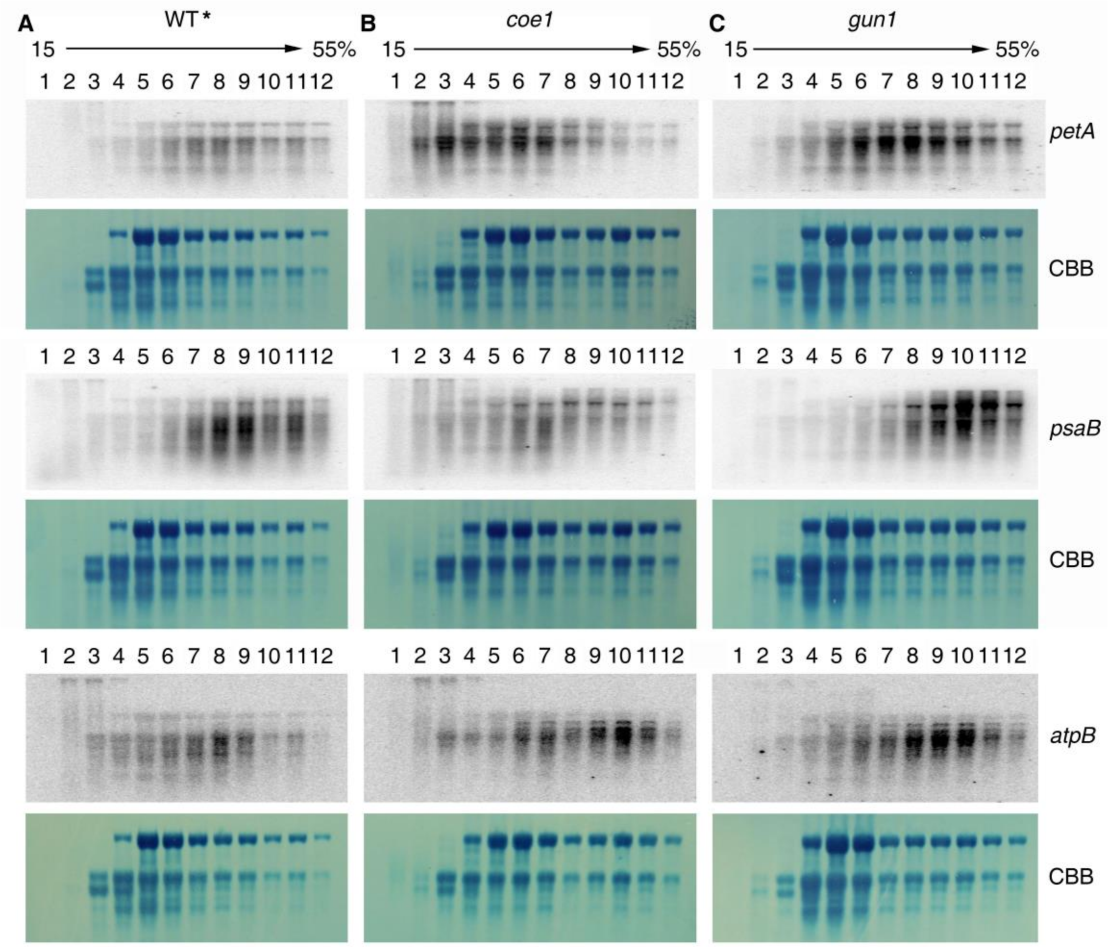

Figure S.7

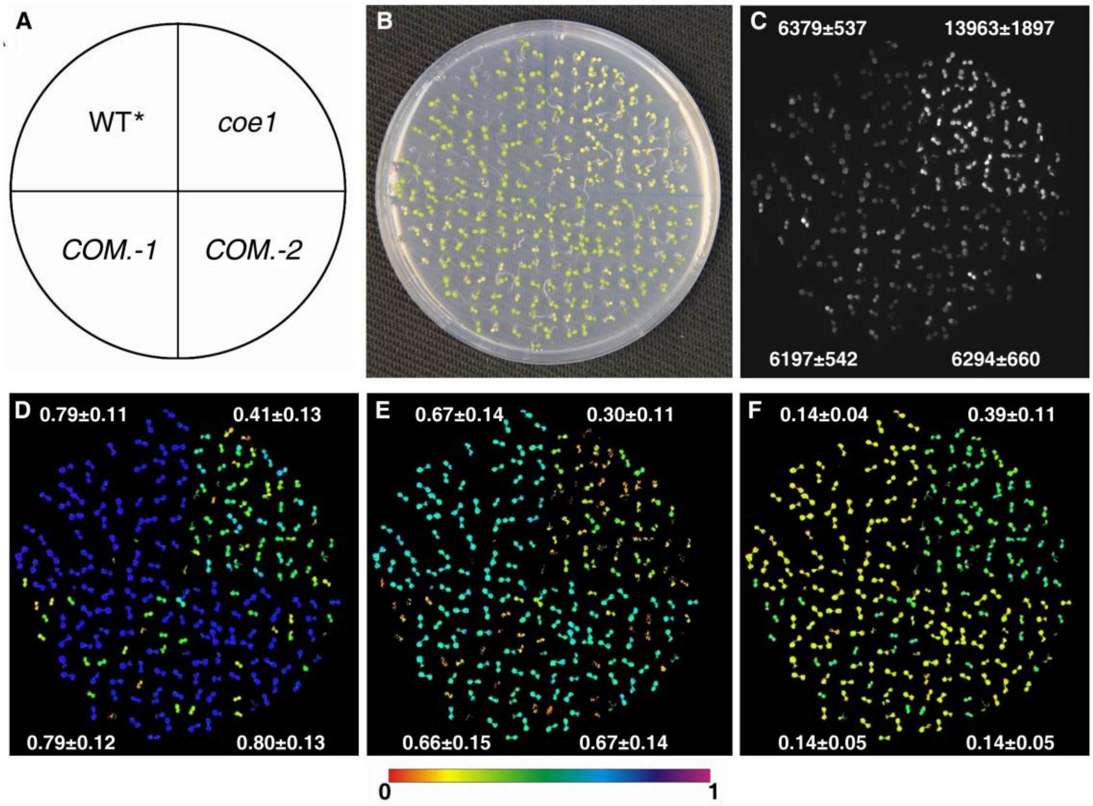
A
WT*
coe1
COM.-1
COM.-2
B
C
6379±537
13963±1897
6197±542
6294±660
D
0.79±0.11
0.41±0.13
0.79±0.12
0.80±0.13
E
0.67±0.14
0.30±0.11
0.66±0.15
0.67±0.14
F
0.14±0.04
0.39±0.11
0.14±0.05
0.14±0.05
0
1

Figure S.8

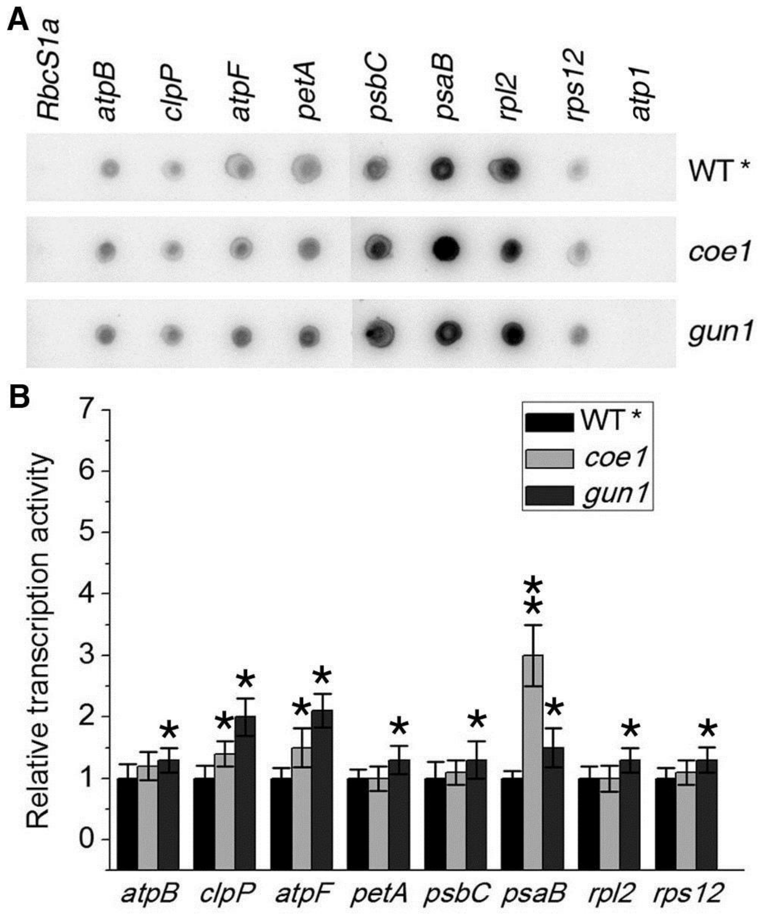
A
RbcS1a
atpB
clpP
atpF
petA
psbC
psaB
rpl2
rps12
atp1
WT *
coe1
gun1
B
Relative transcription activity
0
1
2
3
4
5
6
7
WT *
coe1
gun1
atpB
clpP
atpF
petA
psbC
psaB
rpl2
rps12

Figure S.9

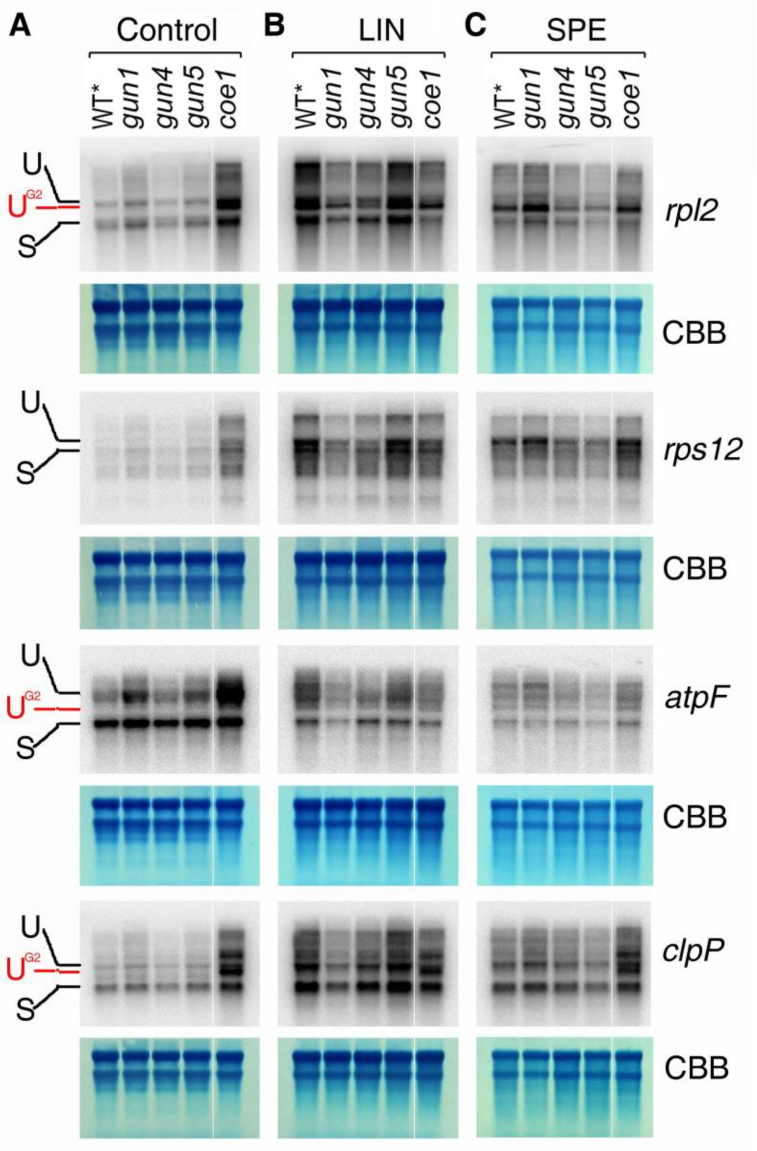

Figure S.10

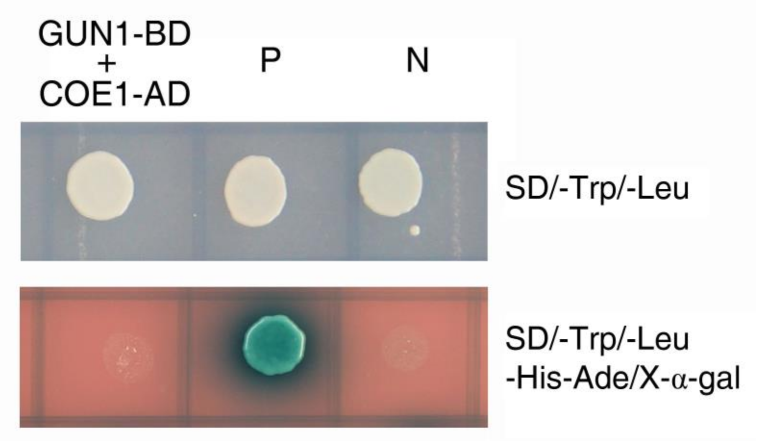

Figure S.11

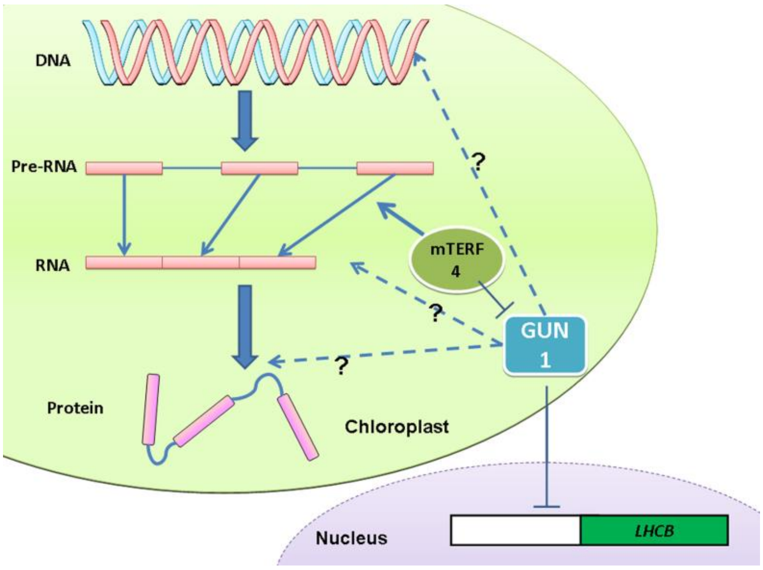
DNA
Pre-RNA
RNA
Protein
mTERF
4
?
?
?
GUN
1
Chloroplast
Nucleus
LHCB

Figure S.12
